# Genome-Mining Based Discovery of Pyrrolomycin K and L from the Termite-Associated *Micromonospora* sp. RB23

**DOI:** 10.1021/acs.jnatprod.5c01051

**Published:** 2025-11-06

**Authors:** Min Lin, Martinus de Kruijff, Michael Poulsen, Christine Beemelmanns

**Affiliations:** † Department Antiinfectives from Microbiota, 443745 Helmholtz Institute for Pharmaceutical Research Saarland (HIPS), 66123 Saarbrücken, Germany; ‡ Section for Ecology and Evolution, Department of Biology, University of Copenhagen, 2100 Copenhagen East, Denmark; § Pharma Science Hub, Saarland University, 66123 Saarbrücken, Germany

## Abstract

Natural products derived from symbiotic microbes remain a rich source of structurally diverse and bioactive molecules. In this study, we report *de novo* genome sequencing of the termite-associated isolate *Micromonospora* sp. RB23. Genome mining uncovered a type I polyketide synthase (T1PKS) biosynthetic gene cluster encoding five halogenases, predicted to produce pyrrolomycin-like antimicrobial compounds. Mass-spectrometry-based molecular networking facilitated the identification and isolation of *N*-methylated pyrrolomycin K and mycothiol-adduct, pyrrolomycin L. Structure elucidation was accomplished based on liquid chromatography high-resolution tandem mass spectrometry (LC-HRMS/MS) alongside 1D and 2D nuclear magnetic resonance (NMR) spectroscopy. Based on the evaluated of antimicrobial activity, we propose that *N*-methylation and mycothiol-based conjugation in pyrrolomycins are possible detoxification mechanisms that play a role in enhancing self-tolerance.

## Graphical abstract

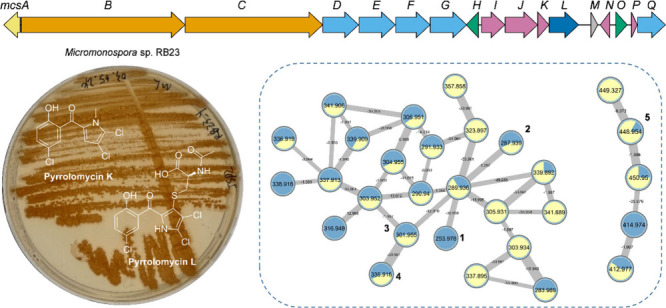

Natural product discovery driven by ecological insights and genome mining has emerged as a powerful strategy for identifying novel chemical scaffolds and biosynthetic chemistry driving their production.^1,2^ Notably, members of the phylum Actinomycetota, particularly those involved in protective symbiosis have been recognized as prolific producers of natural products.^3^ In case of the firebugs (Pyrrhocoridae: *Pyrrhocoris apterus*)^4^ or the European beewolf (Crabronidae: *Philanthus*),^5^ antibiotic-producing *Streptomyces* help protect offspring from fungal infections. Insect-associated Actinomycetota strains have also been reported from other insects, such as fungus-growing ants (*Attini* species) that carry protective *Pseudonocardia* that are active against specialized parasites.^6^ More recently, studies have also shown that dung beetles (Scarabaeidae: *Copris tripartitus*)^7,8^ maintain their offspring in brood balls containing protective *Streptomyces*.^9^ We and others have also shown that in fungus-growing termites (Termitidae: Macrotermitinae), members of the Actinomycetota residing within termite guts and fungus gardens (fungus combs) help protect the termite’s fungal mutualist *Termitomyces* (Basidiomycota: Agaricales: Lyophyllaceae).^2,10,11^ Over the past years, we have analyzed representative Actinomycetota,^12–14^ and more recently also less common, yet bioactive, members of these communities.

Only recently, a *Micromonospora* isolate caught our attention due to its antimicrobial activity. *Micromonospora* is a highly diverse genus of Actinomycetota that thrives in a variety of habitats, including terrestrial soils, marine sediments, and a range of host organisms.^15,16^ Notably, *Micromonospora* is well recognized for the ability to produce structurally diverse and biologically active natural products,^17^ as exemplified by the marine-derived *Micromonospora* sp. WMMA-2495, which was found to produce phallusialides; alkaloid compounds exhibiting activity against methicillin-resistant *Staphylococcus aureus* (MRSA) and *Escherichia coli*.^18^ Additionally, anthraquinones known as lupinacidins, isolated from the endophytic *Micromonospora lupini*, demonstrated anti-invasive effects against tumor cells.^19^

Only a few species in this genus have been investigated in the context of insect symbioses, and even fewer are from termites. Thus, we herein investigated the termite-associated *Micromonospora* sp. RB23 in greater depth to elucidate the basis of its antimicrobial activity.^12^ Building on a newly generated high-quality *de novo* genome sequence of strain RB23, and a comprehensive analysis of its biosynthetic gene cluster (BGC) repertoire, we hypothesized that a predicted halogenated pyrrole-derived natural product could be (at least partially) responsible for the observed antimicrobial activity. This pursuit was motivated by the well-established role of halogens in diverse classes of antibiotics and antimicrobial scaffolds, with approximately 25% of approved pharmaceuticals, such as vancomycin, rebeccamycin,^200^ and pyrrolnitrin^201^ – containing halogen substituents.^20^ Fermentation followed by semitargeted analysis uncovered the production of a pyrrolomycin-derived metabolite family and led to the isolation of two pyrrolomycin derivatives: a rare *N*-methylated pyrrolomycin **3** and an antimicrobial *N*-acetyl-cysteinylated congener **5**.

## Result and Discussion

### Discovery of a Putative Pyrrolomycin-Type BGC via Genome Mining

*Micromonospora* sp. RB23 was isolated from the gut fluids of *Macrotermes natalensis* in a previous study.^3^ To validate its ecological effect, the strain was cocultured with a fungal cultivar *Termitomyces* sp. (strain T153) and the coevolved stowaway fungus *Xylaria* sp. (strain X802). As previously reported, RB23 was weakly inhibitory against both *Termitomyces* sp. T153 as well as *Xylaria* sp. X802 and thus was suspected to modulate growth activities within the fungus comb environment (Figure 1, Figure S1, S2).

**Figure 1:**
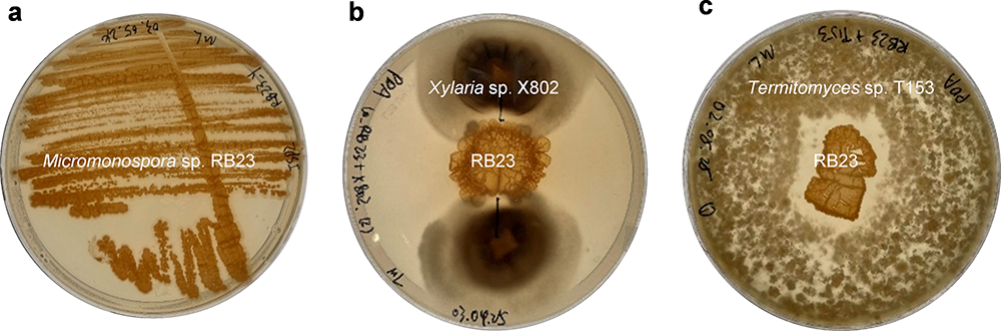
(a) *Micromonospora* sp. RB23 on ISP2 agar, (b) cocultivation of *Micromonospora* sp. RB23 and *Xylaria* sp. X802, and (c) with *Termitomyces* sp. T153.

To investigate whether secondary metabolites contribute to the observed antifungal activity, the genome of the strain was sequenced using Oxford Nanopore Technology. After assembly and annotation, a complete genome of 6.8 Mb in length was obtained with a GC content of 71.8%. Genome-based taxonomic analysis using the Type (Strain) Genome Server (TYGS)^21,22^ placed the strain within a subcluster of the *Micromonospora* genus, identifying *Micromonospora lupini* JCM 16031 ^23^ as its closest known relative (Figure S3), with digital DNA–DNA Hybridization (dDDH) *d*_4_ value (confidence intervals) of 39.2% (36.7–41.7%), and ANI values of 89.3% (72.1%) indicating that RB23 likely represents a new species within the genus.^24,25^

Subsequently, the web-based analyses using antiSMASH v8.0 ^26^ and MIBiG 4.0 ^27^ uncovered about 16 biosynthetic gene cluster regions putatively involved in natural product biosynthesis. Of these, seven were tentatively annotated as terpene or terpene precursor biosynthesis, three BGCs encoded polyketide synthases (PKS), and one cluster region contained a nonribosomal peptide synthetase (NRPS) (Table S3). Among these, the annotated PKS-containing BGC (*mcs*) caught our interest due to the presence of genes annotated as a T1 PKS (*mcsB* and *mcsC*), which was tailored by five genes annotated as different flavin-dependent halogenases (FDHs) (*mcsD*, *E*, *F*, *G*, and *Q*). Cluster blast analysis using the MIBiG database suggested an overall moderate similarity to pyrrolomycins (*pyr*),^28^ marinopyrrole (*mpy*),^29^ pyoluteorin (*plt*),^30^ pyralomicin (*prl*),^31^ and in combination with the difference in the gene cluster architecture, we suspected structural variations and yet unreported metabolite modifications (Figure 2).

**Figure 2:**
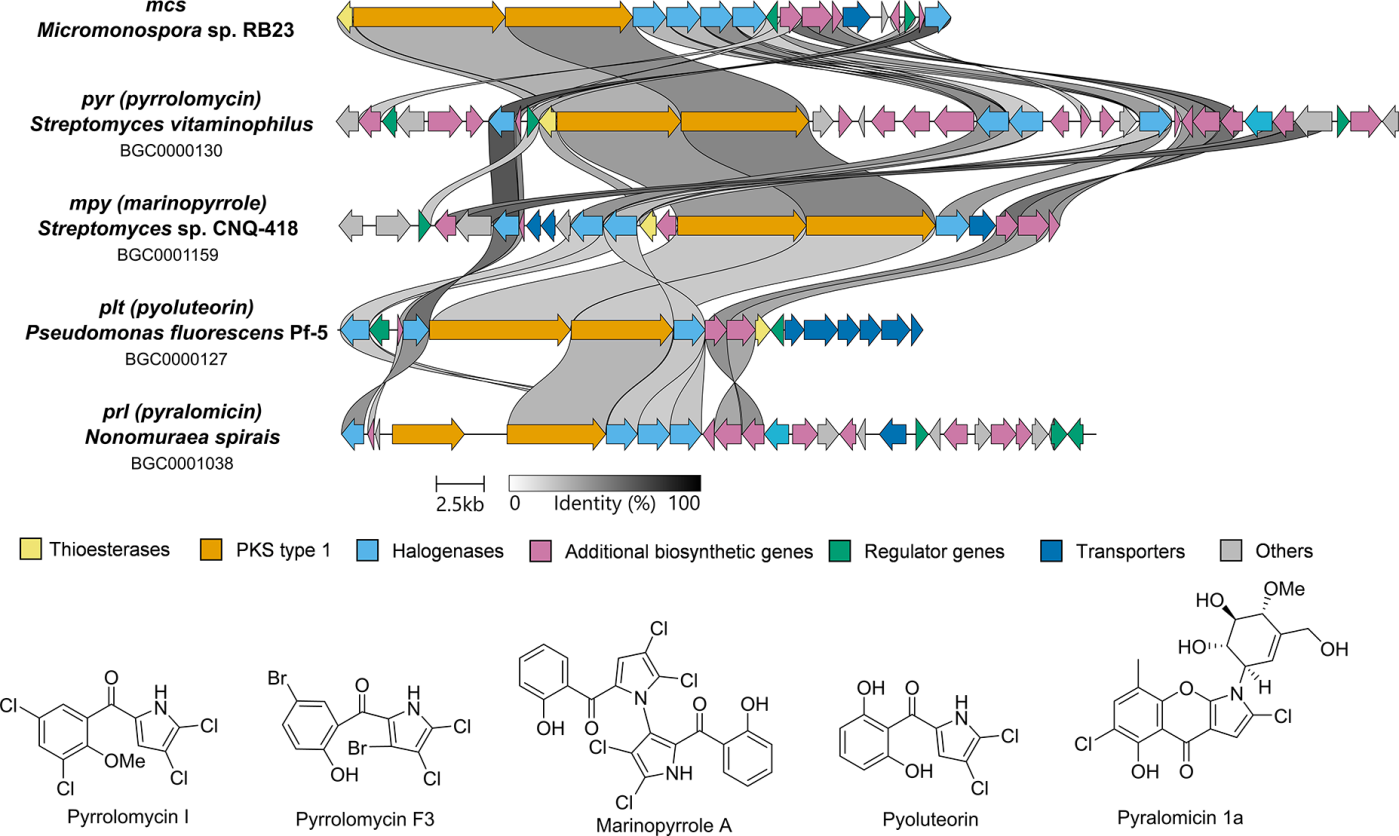
Clinker-based comparison of BGC architectures of *mcs* (pyrrolomycin) encoded in *Micromonospora* sp. RB23, and homologous sequences *pyr* (*Streptomyces vitaminophilus*), *mpy* (*Streptomyces* sp. CNQ-418), *plt* (*Pseudomonas fluorescens* Pf-5), and *prl* (*Nonomuraea spiralis*),^32^ and corresponding pyrrolomycin-type natural products.

### Metabolomic Survey and Detection of Pyrrolomycins

To identify any possible halogenated pyrrol-based metabolites originating from *mcs* biosynthesis, strain RB23 was cultivated on ISP2 agar plates with the addition of 0.5% NaCl at 30 °C for 10 days. After solid phase extraction, the crude extract was dissolved in MeOH and measured using liquid chromatography high-resolution tandem mass spectrometry (LC-HRMS/MS) in both positive and negative mode. Mass spectrometry data was visualized using web-based Global Natural Products Social Molecular Networking (GNPS)^33^ and Cytoscape^34^ (Figure 3a). A closer manual inspection of the molecular ion features uncovered the expected (poly) chlorine-isotope-specific halogenation pattern within two clusters encompassing more than 20 nodes in negative mode. Dereplication of *m*/*z* features was carried out to search against public databased like GNPS, Natural Product Atlas (NPAtlas)^35^ and the Collection of Open Natural Products (COCONUT).^36^ Indeed, one node with the *m*/*z* value of 335.9160 [M – H]^−^ was tentatively annotated as the known halogenated metabolite pyrrolomycin I (Figure 2). Furthermore, we were able to detect additional halogen-containing molecular ion features in low intensity. One feature (*m*/*z* 253.9783) was tentatively annotated as monodeoxypyoluteorin (**1**), which was previously described from *Streptomyces* sp. CNQ-418 as biosynthetic precursor of marinopyrroles (Figure 2).^29^ A second feature (*m*/*z* = 287.9392) was tentatively annotated as compound **2**. While structurally similar to natural product **1**, compound **2** has not been reported from natural sources, but was chemically synthesized and showed potent activity against resistant *S. aureus* and several *Tricophyton* species.^37^ Several related nodes with nearly equal abundance remained unassigned but may feature possible novel derivatives (Figure 3a). Since the detected pyrrolomycin-related molecular ion features contained between one and four chlorine atoms, we examined whether supplementation with KBr (0.05–0.5%) would alter the halogenation pattern. However, no mono- or polybrominated molecular ion features were detected in the MS/MS analysis, even after the addition of up to 0.5% KBr to the culture broth. To isolate some of the “unknown knowns”,^38^ large-scale culture was carried out using both, 2 L ISP2 agar with addition of 0.5% NaCl, as well as 10 L PDB (30 °C). In case of agar-plate based cultivation, the mycelium-covered agar was cut into small pieces and first extracted once with EtOAc and subsequently extracted twice with MeOH. Methanolic extracts were then subjected to solvent-partitioning using equal volume of EtOAc, and the combined EtOAc extract were used for purification. In the case of broth cultivation, the culture supernatant was first collected by centrifugation and then extracted by equal volume of EtOAc three times. Concentrated extracts from both—agar and liquid—cultures, were purified by reverse-phase (C_18_) chromatography to finally yield two congeners, pyrrolomycin K (3.7 mg) and pyrrolomycin L (3.59 mg), which showed a typical UV–vis spectrum (maximum absorption at 192, 224, and 316 nm) consistent with previously isolated pyrrolomycins (Figure 3b,c, Figure S4),^28–31^ but with yet unreported *m*/*z* and NMR patterns.

**Figure 3:**
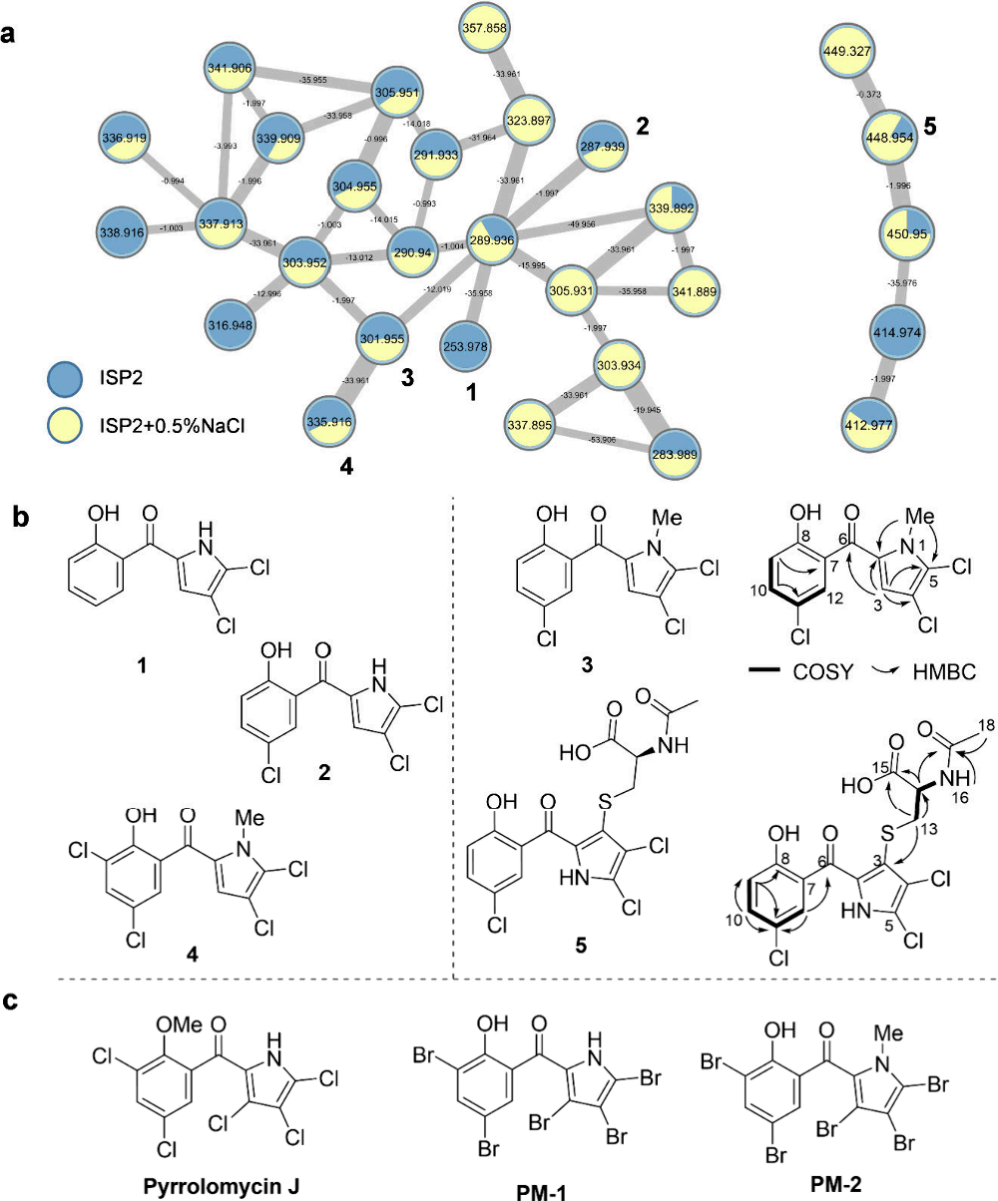
(a) HR-MS^2^ based GNPS clusters (negative ion mode) obtained from plate extracts. Based on dereplication of their *m*/*z* values, five nodes were putatively assigned to monodeoxy pyoluteorin (**1**, *m*/*z* 253.9783 [M – H]^−^), 5′chlorodeoxy-pyoluteorin (**2**, *m*/*z* 287.9392 [M – H]^−^) previously reported from chemical synthesis, pyrrolomycin K (**3**, *m*/*z* 301.9548 [M – H]^−^), pyrrolomycin L (**5**, *m*/*z* 448.9538 [M – H]^−^), and isomer of known derivative pyrrolomycin I (**4**, *m*/*z* 335.9160 [M – H]^−^). Blue nodes represented features from ISP2 plate, while yellow nodes showed features from ISP2 plates containing 0.5% NaCl; (b) chemical structure of **1**–**5** and selective 2D NMR correlations of **3** and **5**; (c) structure of O-methylated pyrrolomycin J and synthetic compounds PM-1 and PM-2.

### Structure Elucidation of Pyrrolomycin K and Pyrrolomycin L

Pyrrolomycin K (**3**) was isolated as a yellow solid, and its molecular formula was determined to be C_12_H_8_Cl_3_NO_2_ (*m*/*z* 301.9548, [M – H]^−^). The diagnostic fragment ions [C_4_H_2_Cl_2_N]^−^ at *m*/*z* 133.9570 confirmed the presence of dichlorinated pyrroles and [C_6_H_4_ClO]^−^ at *m*/*z* 126.9956 for the chlorophenol (Figure S5). For complete assignment, ^1^H and ^13^C NMR analysis was performed in acetone-*d*_6_ (Table S5, Figures S10–S14). The carbonyl C-6 resonates at 186.1 ppm, and the phenolic C-8 appears at 159.7 ppm. Aromatic carbons C-9, C-10, and C-12 showed proton resonances at 7.05, 7.52, and 7.73 ppm with diagnostic *ortho*- (8.8 Hz) and *meta*- (2.8 Hz) coupling constants. The *N*-methyl group C-13 at 34.1 ppm correlated with the corresponding ^1^H singleton at 3.96 ppm. Quaternary carbons at 128.6, 110.1, and 125.1 ppm of the pyrrolic ring were assigned based on the HMBC correlations to the *N*-methyl group and to the C-3 singleton at 6.96 ppm. The remaining quaternary signal at 123.5 ppm was assigned to chlorine-substituted C-11 in the phenol ring. The *N*-methylated pyrrole was further proven by the MS/MS fragment ions [C_5_H_4_Cl_2_N]^−^ at *m*/*z* 147.9726.

Pyrrolomycin L (**5**) was isolated as a dark yellow solid, and its molecular formula was determined to be C_16_H_13_Cl_3_N_2_O_5_S (*m*/*z* 448.9538, [M – H]^−^). The molecular structure was attributed to an N-acetyl-cysteinylation form of compound **2** as it showed the same MS/MS fragment of dichlorinated pyrroles. A neutral loss of 129 Da from [M – H]^−^ 448.9538 to product ion *m*/*z* 319.9112 indicated fragmentation at the linkage to the acetyl-cysteine group (Figure S5). Due to the compound’s poor solubility in acetone, DMSO-*d*_6_ was used for NMR measurement instead (Table S5, Figures S15–S20). The NMR pattern exhibited the typical chemical shift for the pyrrolomycin skeleton but this time did not exhibit the *N*-methylation found in compound **3**. The acetyl-cysteine moiety was deduced to be linked via a thioether to C-3 due to the occurrence of HMBC correlation from H-13 to C-3. The methyl ester was confirmed by the correlation from H-14, H-16 to C-17 and from H-18 to C-17, the free carboxylic acid was assigned by the correlation from H-13, H-14 to C-15. Similar to pyrrolomycin L (**5**) other natural products have been reported to contain *N*-acetyl-cysteine moieties, like *N*-acetyl-cysteinylated streptophenazines and nanaomycin H, the latter carrying both, *N*-acetyl-cysteinylated and mycothiol forms of the pyronaphthoquinone core (Figure S6).^39,40^ These findings suggest that pyrrolomycin L (**5**) likely results from a mycothiol (MSH)-dependent detoxification pathway, catalyzed by a mycothiol S-conjugate amidase (Mca), as commonly observed in Actinomycetota.^41,42^ To determine the configuration of the *N*-acetylcysteine moiety, pyrrolomycin L was subjected to desulfurization using Raney-nickel prior to Marfey analysis (Figure S7).^43^ By comparative analysis, we deduced that compound **5** contains an *N*-acetyl-l-cysteine moiety.

Given the presence of an unusual *N*-methylation on the pyrrolomycin scaffold, we re-examined our MS/MS data to identify additional *N*-methylated pyrrolomycin derivatives. A more detailed analysis of the MS^2^ spectra revealed that the previously annotated, low-abundance molecular ion feature assigned to pyrrolomycin I also contained the corresponding diagnostic fragment ion *m*/*z* 147.9726 corresponding to [C_5_H_4_Cl_2_N]^−^, and the *m*/*z* feature 160.9568 [C_6_H_3_Cl_2_O]^−^ assigned to the nonmethylated dichlorophenol (Figure S5). Accordingly, we inferred that the molecular ion feature was likely erroneously assigned during the initial dereplication process and that feature **4** more likely corresponds to the *N*-methylated regioisomer of pyrrolomycin I.

### Antimicrobial Activity Testing

Pyrrolomycins are characterized by their hybrid chemical architecture, featuring a pyrrole ring linked to a hydroxylated phenol unit through a carbon bridge. Driven by their notable spectrum of bioactivities, including antibacterial, antibiofilm, and anticancer effects, research to date has largely concentrated on their chemical synthesis and biological profiling.^44–48^ The potency of these compounds is closely linked to the degree of halogenation and the specific configuration of the pyrrole core. Structural optimization strategies, such as increasing or substituting halogen atoms or incorporating electron-withdrawing groups like nitro moieties, have been investigated to enhance efficacy while minimizing cytotoxicity.^49,50^ Synthesized compound **2** lacking the *N*-methylation showed potent activity against *S. aureus* (<1.0 μg/mL).^36^ Notably, synthetic compound **PM-1** (Figure 3) carrying five bromine atoms was also highly active against *S. aureus* (IC_50_ = 3.4 nM).^50^ Recent studies have clarified their mode of action, proving that pyrrolomycins act as protonophore to disrupt bacterial proton motive force (PMF).^51^ Marinopyrroles, which are dimers of pyrrolomycins exhibit the same mode of action.^52^ Furthermore, Raimondi et al. suggested that the biological activity may also arise from steric hindrance and narcotic effect from lipophilicity in the context of cellular membrane.^53^

Based on this background, pyrrolomycins K (**3**) and L (**5**) were tested for antimicrobial activity. However, both compounds were active against neither the fungal mutualist (*Termitomyces* spp. T153) nor *Candida albicans* DSM1665. Despite likely being a detoxification-derived product, *N*-acetyl-cysteine adduct **5** showed weak activity against *S. aureus* ATCC 29213 and *E. coli* ΔtolC, while antimicrobial activity was abolished for *N*-methylated compound **3** (Table S6). This supports the hypothesis that the free amine group likely affects the mode of action and that both *N*-methylation and thiolation are likely to be a result of a self-protection mechanism. This phenomenon was also observed in a similar study on methyl-substituted brominated derivative **PM-2** (Figure 3), which showed a much lower minimum bactericidal concentration (MBC) against *S. aureus* and lower cytotoxicity IC_50_.^53^

### Biosynthetic Considerations

We then revisited the biosynthetic gene cluster architecture of the PKS-encoding BGC (*mcs*) that is presumed to be responsible for the production of pyrrolomycins in RB23. The identified *mcs* region spanned approximately 54.6 kbp encoding approximately 37 genes, of which only a subset appeared to be directly involved in the putative biosynthesis (Table S4). Most importantly, two genes *mcsI* and *mcsJ* were tentatively assigned to encode for an adenyltransferase and the pyrrolidine oxidase, respectively, that would participate in construction of the acyl-pyrrole, two genes appeared to have regulatory functions (*mcsH* and *mcsO*), one gene (*mcsL*) encoded for a drug resistance transporter, and one gene (*mcsN*) was putatively annotated as a methyltransferase. Based on the structural assignment and the similarity of the *mcs* gene cluster to other known BGCs *pyr*, *mpy*, *plt*, and *prl*,^28–31^ we propose a tentative biosynthetic pathway (Figure 4).

**Figure 4:**
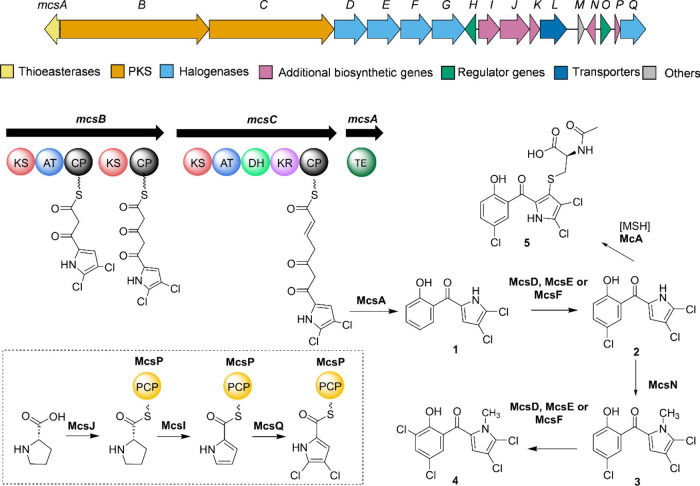
Architecture of putative biosynthetic pathway *mcs* and a proposed biosynthetic assembly line for the formation of pyrrolomycins.

The biosynthesis of pyrrolomycins in RB23 is likely initiated by the modification of PCP-bound l-proline following a sequence of dehydrogenation and halogenation. The PKS-extension of the PCP-bound halogenated intermediate is predicted to be carried out by the PKS modules McsB and McsC. Previous studies have shown that the first KS domain within the PKS module plays a crucial role in determining if the extension can be successfully initiated.^54^ Yi et al. also demonstrated that the first KS domain PltB-KS in the *plt* pathway showed strict substrate specificity for dichlorinated and dibrominated pyrroles, rather than for unsubstituted, monohalogenated, or methylated pyrroles. In contrast, the CalA-KS from calcimycin pathway displays a broader substrate tolerance. Through mutation analysis, they identified a conserved methionine residue Met222 to be essential for the selective extension of dichloropyrroles.

Substitution of methionine with leucine showed a 4-fold increase in pyrrole utilization, while the complementary study on CalA Leu221Met reduced its depletion on the pyrrole substrate. Based on the similarity between McsB and PltB and the presence of a diagnostic methionine motif in McsB, we hypothesized that McsB is likely more substrate selective (Figure S8). The enzyme-bound intermediate is then presumably released by a stand-alone thioesterase McsA to form the intermediate **1** monodeoxypyoluteorin as also reported for marinopyrrole^28^ and pyrrolomycins.^29^ The timing of *N*-methylation of the pyrrole moiety remains an open question. Due to the detection of nonmethylated pyrrolomycin in our metabolomic data sets, we presume that *N*-methylation occurs at a later stage. Following the PKS-mediated chain extension, additional tailoring steps, such as further halogenation and methylation, are likely catalyzed by dedicated tailoring enzymes. Given the potential influence of halogenation on both chain elongation and structural diversity, we conducted a phylogenetic analysis of the halogenases encoded within the *mcs* BGC. MIBiG hits identified all of these enzymes as flavin-dependent halogenases (FDHs). Consistent with their classification, all FDHs contained characteristic conserved motifs: GxGxxG, associated with flavin binding, and WxWxI, which is known to prevent monooxygenase-specific functions.^55^ A phylogenetic tree was constructed together with other characterized FDHs with SAM-dependent halogenase NobA as the outgroup (Figure 5b).

**Figure 5:**
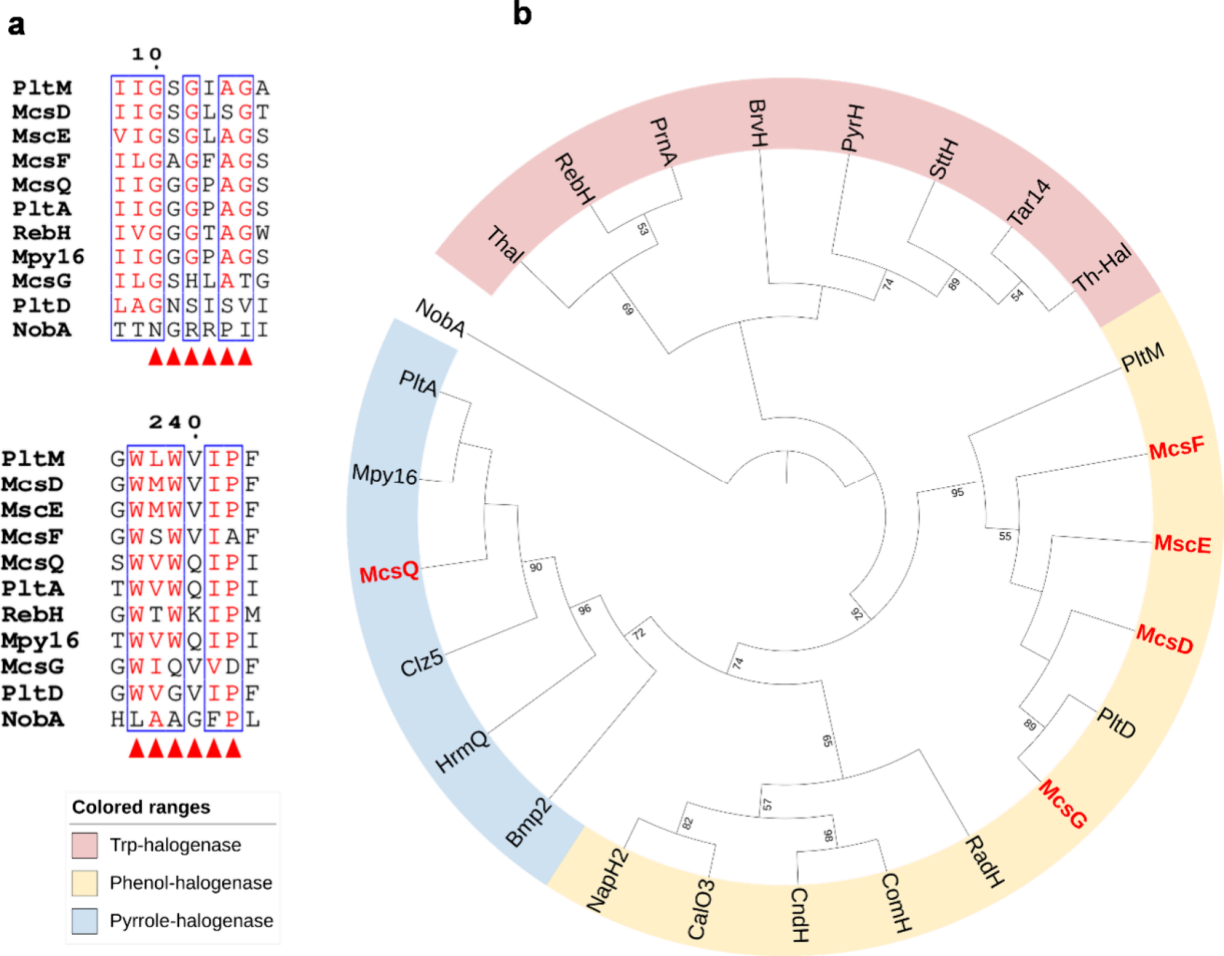
(a) Sequence alignment of FDHs’ conserved motifs GxGxxG and WxWxI. (b) Neighbor-joining phylogenetic tree of the halogenases encoded in the *mcs* BGC (highlighted in bold red), alongside characterized flavin-dependent halogenases (FDHs), using SAM-dependent halogenase NobA as the outgroup. The tree was constructed in MEGA11 ^56^ and visualized using iTOL,^57^ only bootstrap value above 50% (based on 1000 replicates) are shown.

McsQ clustered within the pyrrole-halogenase and shows similarity to the well-studied PltA and Mpy16 known to catalyze the dichlorination of pyrrolyl-S-carrier protein, while McsD, McsE, McsF, and McsG belong to the clade of phenol halogenases and thus are likely to mediate the halogenation of the PKS-derived phenyl ring.^58^ Detailed sequence alignment revealed that McsG, like PltD, lacks key residues within the conserved motifs of FDHs (Figure 5a), suggesting a comparable nonhalogenating function. As reported previously by Yi et al., PltD lacking the conserved FDH motifs, functions as an aromatizing dehydratase rather than a halogenase, catalyzing the formation of dihydro-phloroglucinol during pyoluteorin biosynthesis.^59^

Since the identified structures displayed an unusual *N*-methylation pattern, we also examined the putative methyltransferase McsN encoded within the *mcs* gene cluster. BLAST analysis against the UniProt database combined with Pfam domain annotation revealed homology to phosphatidylethanolamine *N*-methyltransferase (PEMT),^60,61^ an enzyme typically associated with primary metabolism and phosphatidylcholine biosynthesis.^62^ Whether such an enzyme can catalyze the *N*-methylation of a pyrrole moiety remains uncertain. Alternatively, the modification could be introduced by a methyltransferase encoded elsewhere in the genome but with insufficient sequence similarity to known *N*-methyltransferases for reliable identification.

Lastly, we hypothesized that *N*-acetyl-cysteinylated derivative **5** is likely a result of mycothiolation-based detoxification to alleviate redox stress and facilitate detoxification. A BLAST search against the genome of *Micromonospora* sp. RB23 using the well-studied *mca* gene identified from *Mycobacterium tuberculosis* H37Rv^41^ revealed a putative homologue sharing 60% sequence identity. Although located far from the *mcs* cluster (Figure S9), we suspect it may be involved in mycothiolation-mediated detoxification and self-protection.

## Conclusions

In this study, we reported the genome mining-guided discovery and characterization of two pyrrolomycin derivatives, the *N*-methylated pyrrolomycin K (**3**) and an *N*-acetyl-cysteine conjugate pyrrolomycin L (**5**) from *Micromonospora* sp. RB23 is paired with previously reported member monodeoxypyoluteorin (**1**), a chemically synthesized 5′-chlorodeoxy-pyoluteorin (**2**) and potentially unreported *N*-methyl variation (**4**). Comprehensive gene cluster analysis identified a BGC region harboring the core PKS and pyrrole biosynthetic elements. Notably, the presence of multiple halogenase-encoding genes points to a high potential for structural diversification and opportunities in metabolic engineering. Features such as *N*-methylation and mycothiol-based conjugation illustrate possible mechanisms of secondary metabolite detoxification and role in enhancing self-tolerance against harmful compounds. Collectively, these findings broaden the known chemical diversity of pyrrolomycins and the self-resistance strategies employed by the producing *Micromonopsora* isolate.

## Experimental Section

### General Experimental Procedures

NMR spectra were recorded on a Bruker AVANCE III spectrometer operating at 500 MHz (^1^H) and 125 MHz (^13^C) with chemical shift given in parts per million (ppm). UHPLC-HRMS measurement was performed on a Vanquish Flex UHPLC system combined with a Orbitrap Exploris 120 mass spectrometer coupled with a Kinetex C18 column (50 mm × 2.1 mm, particle size 1.7 μm, 100Å, Phenomenex) using mobile phases acetonitrile and H_2_O with 0.1% formic acid. Solid phase extraction was carried out in a CHROMABOND C18 column (45 μm, 6 mL/1000 mg). Preparative HPLC was performed on a Büchi Pure C-835 Prep using a Phenomenex Luna Phenyl-Hexyl 250 mm × 21.2 mm column (particle size 5 μm, pore diameter 100 Å). Semipreparative HPLC was performed on a Vanquish HPLC system using a Phenomenex Luna C18(2) 250 mm × 10 mm column (particle size 5 μm, pore diameter 100 Å) coupled with a diode array detector.

### Genome Sequencing

Whole genome sequencing of strain *Micromonospora* sp. RB23 was performed using Oxford Nanopore sequencing (Oxford Nanopore Technologies, Oxford, UK). The strain was cultivated for 7 days in ISP2 liquid medium, the cell pellet was harvested after spinning down, and the gDNA was extracted using Zymo Research Quick-DNA HMW Magbead Kit according to the manufacturer’s manual. The sequencing run was performed on MinION device using R10.4 flow cell for 48 h. SUP base calling was initially performed with Guppy (v7.1.4) in MinKNOW (v23.07.12), and the data later were rebasecalled with Dorado v0.4.3. Quality control of base-called reads as well as genome assembly was performed with the Hybracter (v0.6.0)^63^ assembly pipeline, applying a minimum read length cutoff of 1kb and retaining top 90% high-quality reads.^64–66^ The genome was annotated using PROKKA (v1.14.5)^67^ and analyzed with QUAST (v5.2.0),^68^ showing a genome size of 6.8 Mbp and a GC content 71.77%. Taxonomy classification was performed with GTDB-Tk (v2.3.2). Average nucleotide identity (ANI) was calculated using the web service available at JSpeciesWS v5.0.2.^69^ The genome was then submitted to antiSMASH (v8.0) using relaxed detection settings to detect the secondary metabolites BGCs within the strain.^26^

### Co-cultivation

Co-cultivation of *Micromonospora* sp. RB23 with *Xylaria* sp. X802 was carried out on PDA plates. RB23 was first cultivated on ISP2 plate for 5 days, and later the mycelium was scratched from the surface and collected in sterile PDB liquid medium to make nearly homogeneous suspension by vortex. Then 20 μL of inoculum was spotted in the center of a new PDA Petri dish (9 cm diameter), which was subsequently cultured at 30 °C for 5 days. Afterward, a fresh *Xylaria* sp. X802 agar plug (1 cm × 1 cm) was cut from a previously prepared PDA plate and was placed next to the RB23 colony with the distance of 1 cm. Co-cultivation was monitored for 2 weeks, axenic cultures of *Micromonospora* sp. RB23 and *Xylaria* sp. X802 were cultivated for the same period as the controls. For the cocultivation of *Micromonospora* sp. RB23 and *Termitomyces* sp. T153, the first mycelium of *Termitomyces* sp. T153 was scratched from a fresh PDA plate and was homogenized by collecting in sterile PDB media and vortex. Then 200 μL of T153 mycelium suspension was placed onto a new PDA Petri dish and evenly distributed by a spreader. Mycelium suspension of *Micromonospora* sp. RB23 was prepared similarly as with X802, and then 20 μL of inoculum was placed into the middle of the PDA plate on the same day. Cultivation was further extended for more than 2 weeks, axenic cultures of *Micromonospora* sp. RB23 and *Termitomyces* sp. T153 were cultivated for the same time period as the controls.

### Cultivation on Agar Plates and Metabolite Extraction

*Micromonospora* sp. RB23 was cultivated in 30 mL of ISP2 media for 5–7 days as seed culture. Plates were inoculated using 100 μL of seed culture and then cultivated at 30 °C for 10 days. Mycelium covered agar plates were cut into small pieces and extracted twice with MeOH. The organic extracts were combined and concentrated under reduced vacuum condition. The crude extracts were resuspended with 20% MeOH (6 mL) and separated by a pre-equilibrated SPE-C18 cartridge (6 mL/1000 mg). Metabolites were eluted by first washing the loaded column with 20% MeOH (10 mL), followed by flushing with 50% MeOH (10 mL) and then 100% MeOH (10 mL). Both fractions were combined and concentrated under vacuum. The resultant extract was dissolved in MeOH (0.1 mg/mL) and submitted to LC-HRMS/MS analysis under both positive and negative mode.

### UHPLC-HESI-HRMS Analysis

UHPLC-HESI-HRMS measurements were carried out on a Vanquish Flex UHPLC system (Thermo Scientific) combined with an Orbitrap Exploris 120 mass spectrometer (Thermo Scientific) equipped with a heated electrospray ionization (HESI) source. Metabolites were separated using reverse phase liquid chromatography at 40 °C using a Kinetex C18 column (50 mm × 2.1 mm, particle size 1.7 μm, 100 Å, Phenomenex) preceded by a C18 SecurityGuardTM ULTRA guard cartridge (2.1 mm, Phenomenex). Mobile phases consisted of H_2_O + 0.1% formic acid (buffer A1) and acetonitrile + 0.1% formic acid (buffer B1) were used for positive mode while mobile phases consisted of H_2_O (buffer A2) and acetonitrile (buffer B2) were used for negative mode. The sample was measured (5 μL, 0.1 mg/mL) and was analyzed using a gradient as follows: 0–1 min, 5% B; 1–10 min, 97% B; 10–12 min, 97% B; 12–13 min, 5% B; 13–15 min, 5% B at a constant flow rate of 0.3 mL/min.

### GNPS Molecular Networking

Raw metabolomic data was converted to 32-bit mzML file using MSconvert GUI (ProteoWizard) and subsequently submitted to the Global Natural Products Social Molecular Networking (GNPS) platform for creating a mass spectral molecular network.^70,71^ Default settings were used for analysis, except the precursor ion mass tolerance was set to 0.02 Da and an MS/MS fragment ion tolerance of 0.02 Da, while edges were filtered to have a cosine score above 0.7 and more than 4 matched peaks. Spectral networks were visualized using Cytoscape 3.10.0.^72^

### Isolation of Pyrrolomycins

For the cultivation performed on ISP2 agar plate with 0.5% NaCl (total volume of 2 L), the agar was harvested after 10 days at 30 °C by cutting into small pieces and extracted by EtOAc once and then MeOH twice; the crude extract was dried under vacuum. The MeOH extract was then dissolved in H_2_O and extracted with equal volume of EtOAc for three times, the resulting extract was combined with the first EtOAc fraction. For cultivation performed on a fermenter with 10 L of PDB medium, the temperature was set at 30 °C with 30% oxygen. After 9 days, the culture was first collected by centrifugation at 6000 rpm for 15 min. The resulting supernatant was extracted by equal volume of EtOAc three times and dried under vacuum. Both extracts from agar and liquid cultures were further fractionized by Büchi Pure C-835 Prep using a Phenomenex Luna Phenyl-Hexyl 250 mm × 21.2 mm column (particle size 5 μm, pore diameter 100 Å) from 10% acetonitrile to 100% acetonitrile at flow rate 20 mL/min for 40 min to afford subfractions F1–F7. Subfraction F7 was separated in a Vanquish HPLC system using a Phenomenex Luna C18(2) 250 mm × 10 mm column (particle size 5 μm, pore diameter 100 Å) using the following conditions: 0–5 min, 70% B; 5–25 min, 70%–100% B, 25–30 min, 100% B, 30–31 min, 100%–70% B, 31–38 min, 70% B (A, H_2_O + 0.1% formic acid; B, CH_3_CN + 0.1% formic acid), with a flow rate of 3 mL/min to obtain pyrrolomycin K (**3**, 3.7 mg). Subfraction F5 was separated using the following conditions: 0–5 min, 40% B; 5–24 min, 40%–60% B; 24–25 min, 60%–100% B; 25–30 min, 100% B; 30–31 min, 100%–40% B; 31–38 min, 40% B (A, H_2_O + 0.1% formic acid; B, CH_3_CN + 0.1% formic acid), with a flow rate of 3 mL/min to obtain pyrrolomycin L (**5**, 3.59 mg).

#### Pyrrolomycin K (**3**)

Amorphous, yellow powder; UV (MeOH) λ_max_ (log ε) 198.0 (1.64), 224.5 (1.76), 316.5 (1.59) nm; ^1^H (acetone-*d*_6_, 500 MHz) and ^13^C data (acetone-*d*_6_, 125 MHz) spectra, see Table S5, Figures S10–14; LC-HRESIMS *m*/*z* 301.9548 [M – H]^−^ (calcd for C_12_H_7_Cl_3_NO_2_, 301.9547)

#### Pyrrolomycin L (**5**)

Dark yellow solid; [α]^22^_D_ −32.9 (*c* 0.30, CH_3_OH); UV (MeOH) λ_max_ (log *ε*) 208.0 (3.16), 322.5 (1.33) nm; ^1^H (DMSO-*d*_6_, 500 MHz) and ^13^C data (DMSO-*d*_6_, 125 MHz) spectra, Table S5, Figures S15–20; LC-HRESIMS *m*/*z* 448.9538 [M – H]^−^ (calcd for C_16_H_12_Cl_3_N_2_O_5_S, 448.9538)

### Absolute Configuration of *N*-Acetyl-cysteine Moiety of Pyrrolomycin L (**5**)

Advanced Marfey’s analyses with Raney nickel was applied to obtain the absolute configuration of the *N*-acetyl-cysteine moiety of **5**. Raney nickel (5.0 mg) was added to the solution of **5** (0.2 mg) in MeOH (0.2 mL), and the mixture was incubated at room temperature overnight and purged with Argon. On the next day, the suspension was filtered and washed with MeOH (1.0 mL × 3) and H_2_O (1.0 mL × 3). The filtrate was concentrated in vacuo and then dissolved in 500 μL of 6 M hydrochloric acid to hydrolyze the acetyl moiety of *N*-acetyl-cysteine, followed by heat treatment at 100 °C for 16 h. The product was concentrated in vacuo, then the residue was dissolved in 100 μL H_2_O. Then 20 μL of 1 M NaHCO_3_ and 50 μL of *N*α-(2,4-dinitro-5-fluorophenyl)-l-valinamide (l-FDVA) were added to 50 μL of hydrolysate, followed by incubation at 37 °C for 1 h. The mixture was neutralized by the addition of 20 μL of 1 M HCl and then concentrated into dryness in vacuo. The resulting dried residue was dissolved in 1 mL of acetonitrile, centrifuge at 13000 rpm for 15 min. Similarly, standard l-alanine and d-alanine were derivatized as described above. The l-FDVA derivatives were subjected to the UHPLC-HESI-HRMS mentioned above using the following gradient program: 0–1 min, 5% B; 1–10 min, 100% B; 10–14 min, 100% B; 14–15 min, 5% B; and 15–17 min, 5% B at a constant flow rate of 0.3 mL/min.

## Supplementary Material



## Data Availability

Raw 1D and 2D NMR files, gbk files of gene cluster and NMR tables have been deposited on the Zenodo repository (10.5281/zenodo.16932486). NMR data has also been uploaded to the NP-MRD database (https://np-mrd.org/) for pyrrolomycin K (NP0351647) and pyrrolomycin L (NP0351648). The genome sequence of *Micromonospora* sp. RB23 is available at GenBank (BioProject, PRJNA1285835; BioSample, SAMN49776535; SRA, SRR34395231). HRMS/MS data (identifier MSV000098869) has been deposited on the MassIVE repository (https://massive.ucsd.edu/).

## Notes

Additional information about experimental procedures and bioinformatic analyses, copies of the 1D and 2D NMR spectra of compound **3** and **5**, and copies of HRESIMS/MS spectra of compounds **1**–**5** (PDF)
