# Supplementary material for: Genome-Mining Based Discovery of Pyrrolomycin K and L from the Termite-Associated Micromonospora sp. RB23
Source: J Nat Prod. 2025 Nov 6;88(11):2701–9. doi: 10.1021/acs.jnatprod.5c01051 (PMC12670696; doi:10.1021/acs.jnatprod.5c01051)
Supplement: Supplementary file 1 [file np5c01051_si_001.pdf]

Supporting Information

Genome-mining based discovery of pyrrolomycin K and L from the termite-associated *Micromonospora* sp. RB23

Min Lin[1], Martinus de Kruijff[1], Michael Poulsen[3], Christine Beemelmanns[1,2]*

[1] Department Antiinfectives from Microbiota, Helmholtz Institute for Pharmaceutical Research Saarland (HIPS), 66123 Saarbrücken, Germany

[2] Pharma Science Hub, Saarland University, 66123 Saarbrücken, Germany

[3] Section for Ecology and Evolution, Department of Biology, University of Copenhagen, 2100 Copenhagen East, Denmark

*corresponding author:

christine.beemelmanns@helmholtz-hips.de

Table of contents

**Figure S1**	*Micromonospora* sp. RB23 co-cultivated with *Xylaria* sp. X802 with axenic cultures served as controls	P5
**Figure S2**	*Micromonospora* sp. RB23 co-cultivated with *Termitomyces* sp. T153 with axenic cultures served as controls	P5
**Figure S3**	Phylogenetic placement of *Micromonospora* sp. RB23	P6
**Figure S4**	Chemical structure of selective natural pyrrolomycins	P8
**Figure S5**	LC-MS/MS spectra of **1-5**	P9
**Figure S6**	Structures of previously reported mycothiol-conjugated compounds	P9
**Figure S7**	Determination of absolute configuration of the N-acetylcysteine moiety derived from compound **5**	P10
**Figure S8**	KS sequence alignment with sequence for pyoluteorin, armeniaspirol, chlorizidine, marinopyrrole, pyralomicin, pyrronazol B, calcimycin and DKxanthene and selected structures	P11
**Figure S9**	Blast result of putative *mca*-like gene in the genome of *Micromonospora* sp. RB23 using mycothiol S-conjugated amidase (Mca) from *Mycobacterium tuberculosis H37Rv* as query	P11
**Figure S10**	^{1}H NMR spectrum of pyrrolomycin K (**3**) in acetone-d_6	P15
**Figure S11**	^{13}C NMR spectrum of pyrrolomycin K (**3**) in acetone-d_6	P16
**Figure S12**	COSY spectrum of pyrrolomycin K (**3**) in acetone-d_6	P17
**Figure S13**	HSQC spectrum of pyrrolomycin K (**3**) in acetone-d_6	P18
**Figure S14**	HMBC spectrum of pyrrolomycin K (**3**) in acetone-d_6	P19
**Figure S15**	^{1}H NMR spectrum of pyrrolomycin L (**5**) in DMSO-d_6	P20
**Figure S16**	^{13}C NMR spectrum of pyrrolomycin L (**5**) in DMSO-d_6	P21
**Figure S17**	COSY spectrum of pyrrolomycin L (**5**) in DMSO-d_6	P22
**Figure S18**	HSQC spectrum of pyrrolomycin L (**5**) in DMSO-d_6	P23
**Figure S19**	HMBC spectrum of pyrrolomycin L (**5**) in DMSO-d_6	P24
**Figure S20**	NOESY spectrum of pyrrolomycin L (**5**) in DMSO-d_6	P25
**Table S1**	Medium compositions used in this study	P4

**Table S2**	GenBank/IMG OID accession numbers of the strains used in Figure S3	P7
**Table S3**	BGCs annotation using antiSMASH 8.0 with relaxed setting	P12
**Table S4**	Proteins encoded in *mcs* BGC and blast result against NCBI Refseq database	P13
**Table S5**	NMR spectroscopic data (500 MHz) of pyrrolomycin K (**3**) and pyrrolomycin L (**5**)	P14
**Table S6**	Results of antimicrobial activity assays	P26

Table S1. Medium compositions used in this study

Medium	Composition (per L)
ISP2 broth/agar	4.0 g yeast extract, 10.0 g malt extract, 4.0 g dextrose, for agar : 2.0% agar (w/v)
ISP2 broth/agar with 0.5% NaCl	4.0 g yeast extract, 10.0 g malt extract, 4.0 g dextrose, 5 g NaCl, for agar : 2.0% agar (w/v)
ISP2 broth/agar with 0.05-0.5% KBr	4.0 g yeast extract, 10.0 g malt extract, 4.0 g dextrose, 0.5-5.0 g KBr, for agar : 2.0% agar (w/v)
PDB/ PDA	26.5 g potato extract glucose broth, for agar: 2.0% agar (w/v)

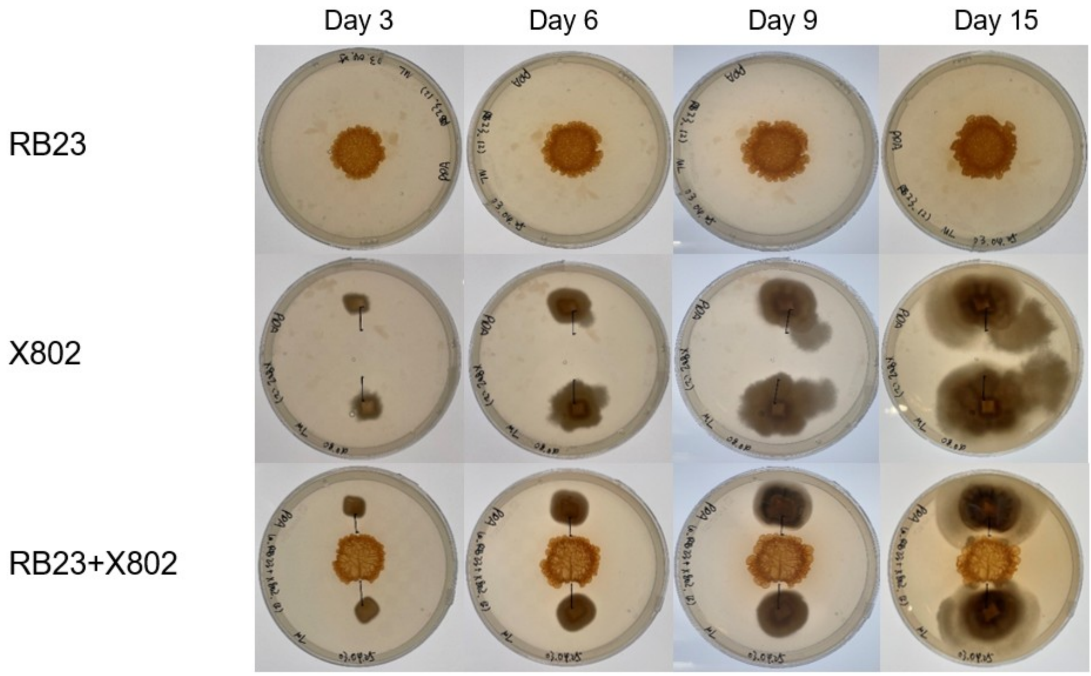

Figure S1. *Micromonospora* sp. RB23 co-cultivated with *Xylaria* sp. X802 with axenic cultures served as controls.

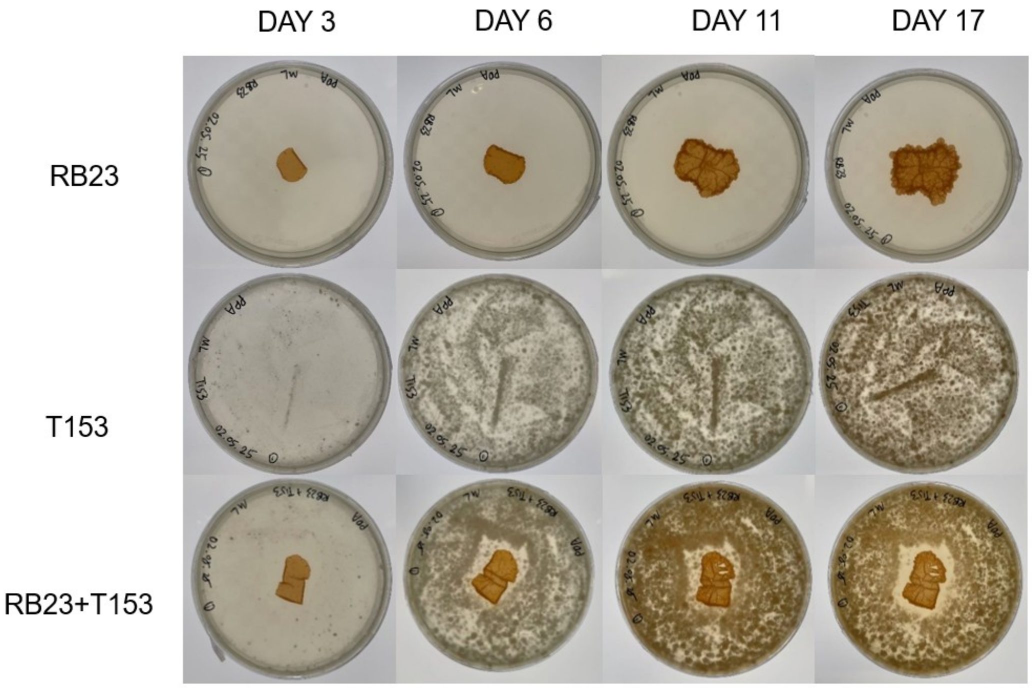

Figure S2. *Micromonospora* sp. RB23 co-cultivated with *Termitomyces* sp. T153 with axenic cultures served as controls.

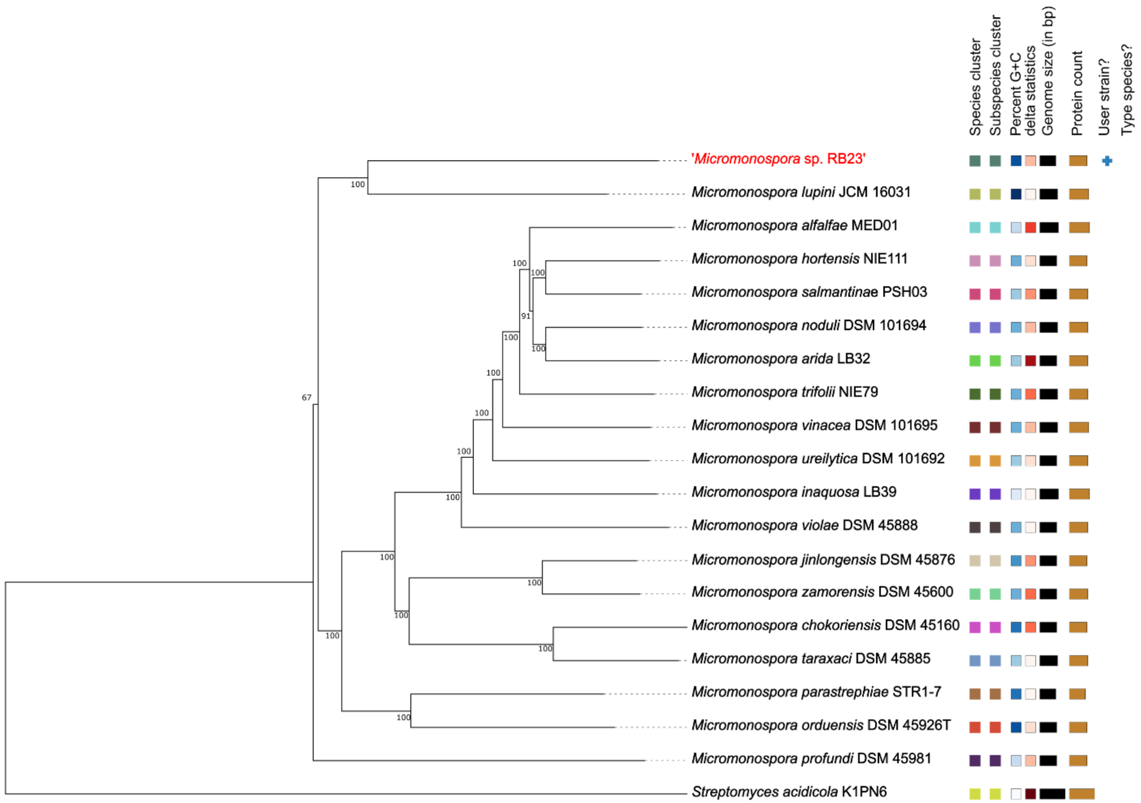

Figure S3. Phylogenetic placement of *Micromonospora* sp. RB23. Tree inferred with FastME 2.1.6.1.[1] from GBDP distances calculated from genome sequences. The numbers above branches are GBDP pseudo-bootstrap support values > 60 % from 100 replications, with an average branch support of 93.5 %. The tree was rooted at the midpoint.[2] *Streptomyces acidicola* K1PN6 was used as outgroup.[3]

Table S2. GenBank accession numbers of the strains used in Figure S3 for the phylogenetic tree analysis, those without NCBI accession numbers, IMG OID that deposit in Integrated Microbial Genomes (IMG) were shown instead.

Strains	Assembly accession	IMG OID
Micromonospora lupini JCM 16031	GCA_039531855	
Micromonospora alfalfa MED01	GCA_022230925	
Micromonospora hortensis NIE111	GCA_022230935	
Micromonospora salmantinae PSH03	GCA_022230905	
Micromonospora noduli DSM 101694	GCA_003264365	
Micromonospora arida LB32	GCA_003857035	
Micromonospora trifolii NIE79	GCA_022229005	
Micromonospora vinacea DSM 101695		2880495981
Micromonospora ureilytica DSM 101692		2880489317
Micromonospora inaquosa LB39	GCA_003857055	
Micromonospora violae DSM 45888	GCA_004217135	2806310543
Micromonospora jinlongensis DSM 45876		2856481432
Micromonospora zamorensis DSM 45600	GCA_900090275	
Micromonospora chokoriensis DSM 45160	GCA_900091505	2623620610
Micromonospora taraxaci DSM 45885	GCA_007830095	2818991475
Micromonospora parastrephiae STR1-7	GCA_016802865	
Micromonospora orduensis DSM 45926T	GCA_006228125	
Micromonospora profundi DSM 45981	GCA_011927785	2830928545
Streptomyces acidicola K1PN6	GCA_009377235	

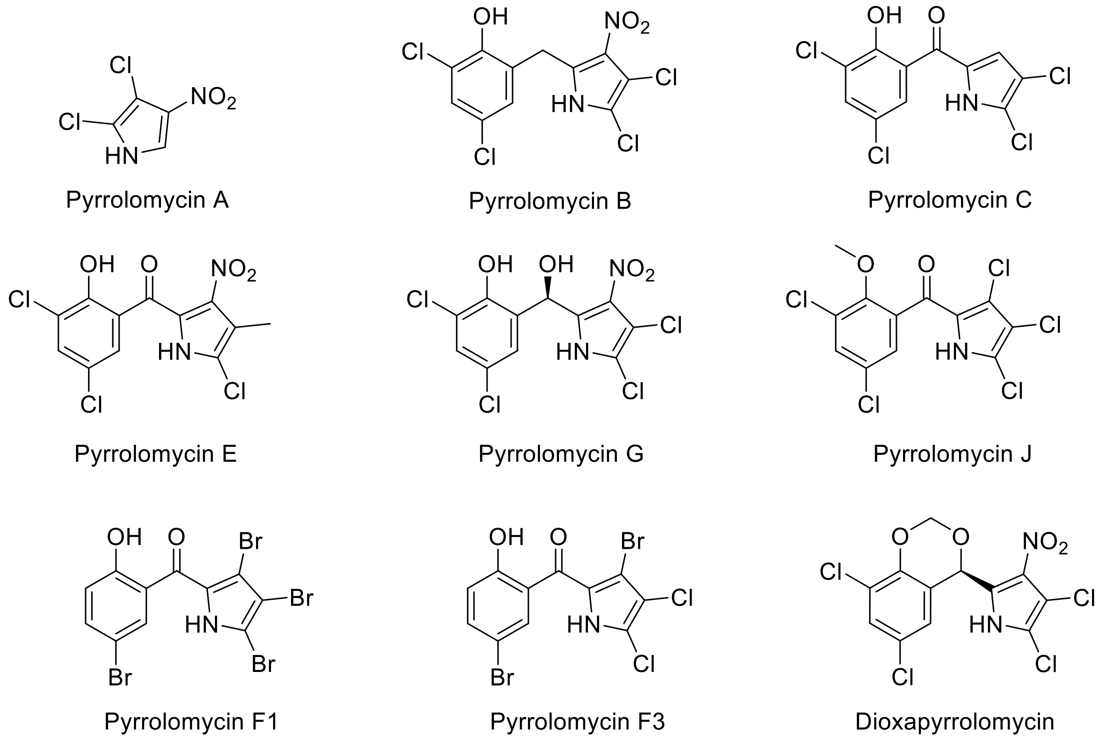

Figure S4. Chemical structure of selective natural pyrrolomycins.[6-10]

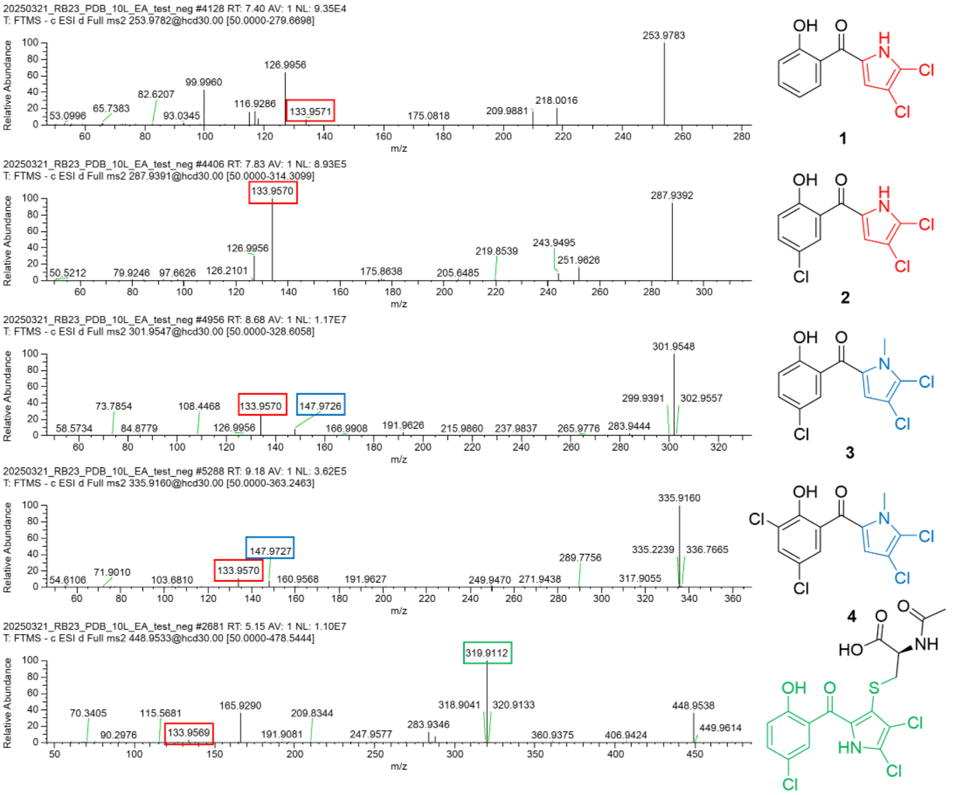

Figure S5. LC-MS/MS spectra of pyrrlomycins (**1-5**) and deduced structures of compound **1**, **2** and **4** based on diagnostic fragments.

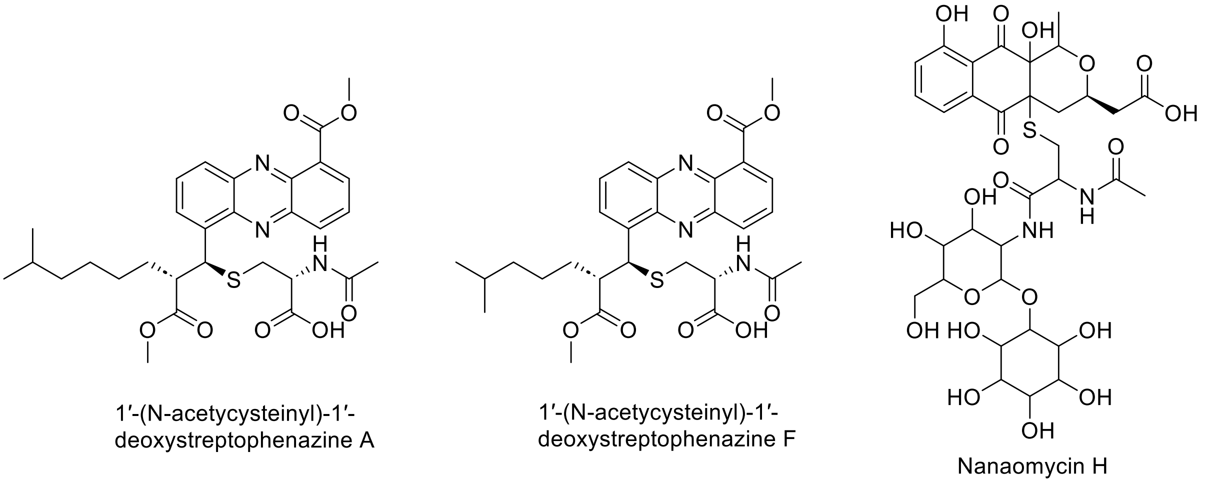

Figure S6. Structures of previously reported mycothiol-conjugated compounds.[4, 5]

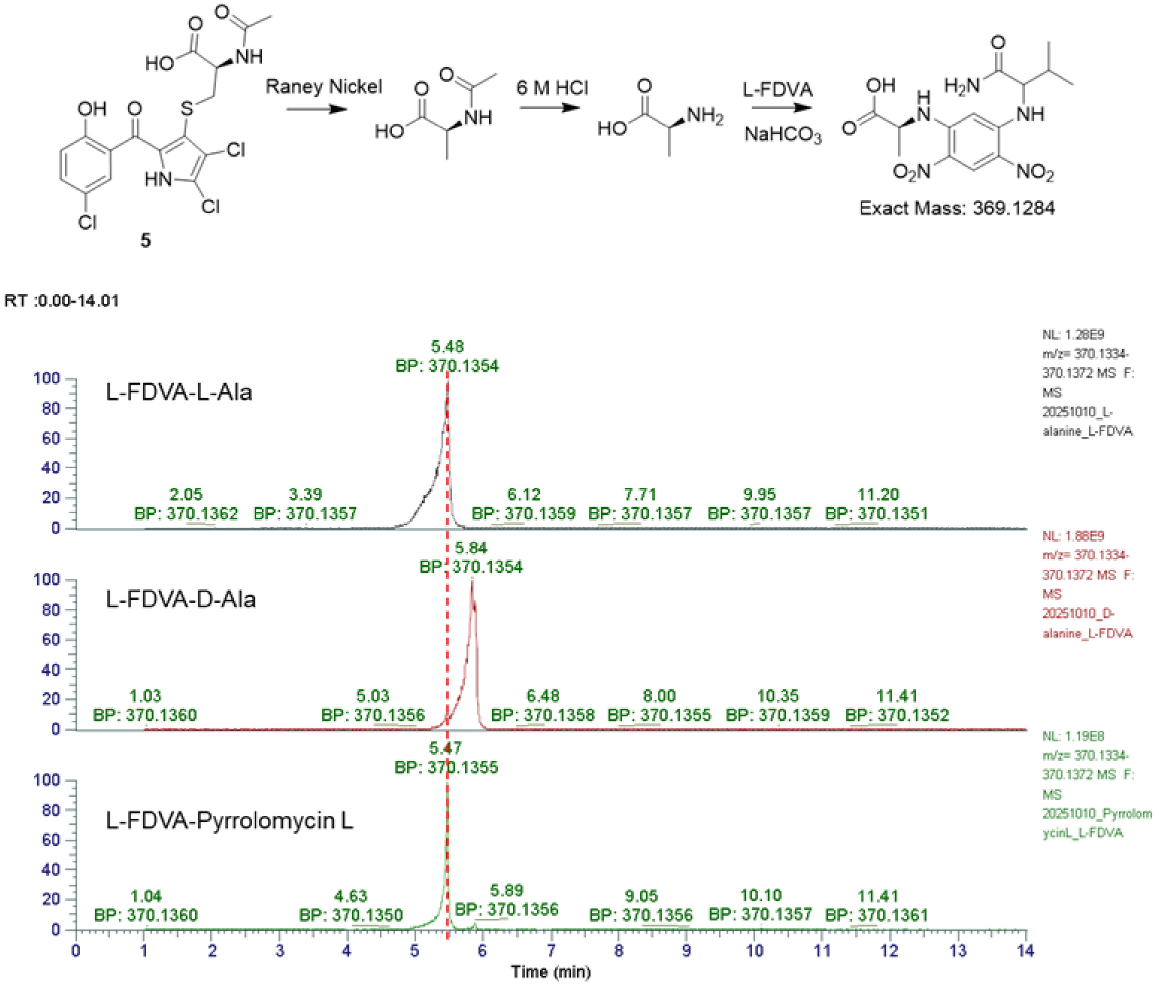

Figure S7. Determination of absolute configuration of the N-acetylcysteine moiety derived from compound 5 using Marfey's method and commercial D- and L-alanine as control.

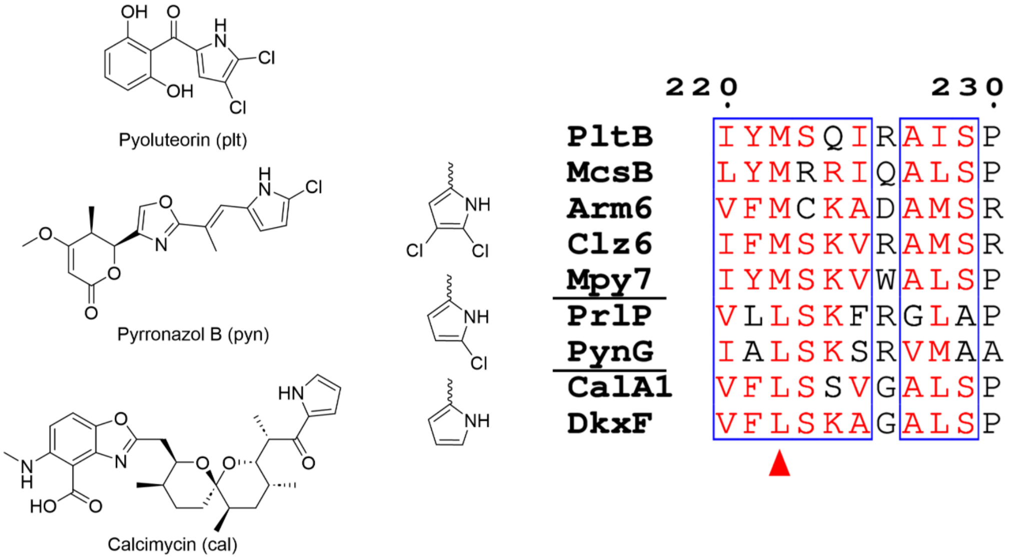

Figure S8. Sequence alignment of KS domains compared with those from the biosynthetic pathways of pyoluteorin, armeniaspirol, chlorizidine, marinopyrrole, pyralomicin, pyrronazol B, calcimycin, and DKxanthene, with representative structures shown.[11-19]

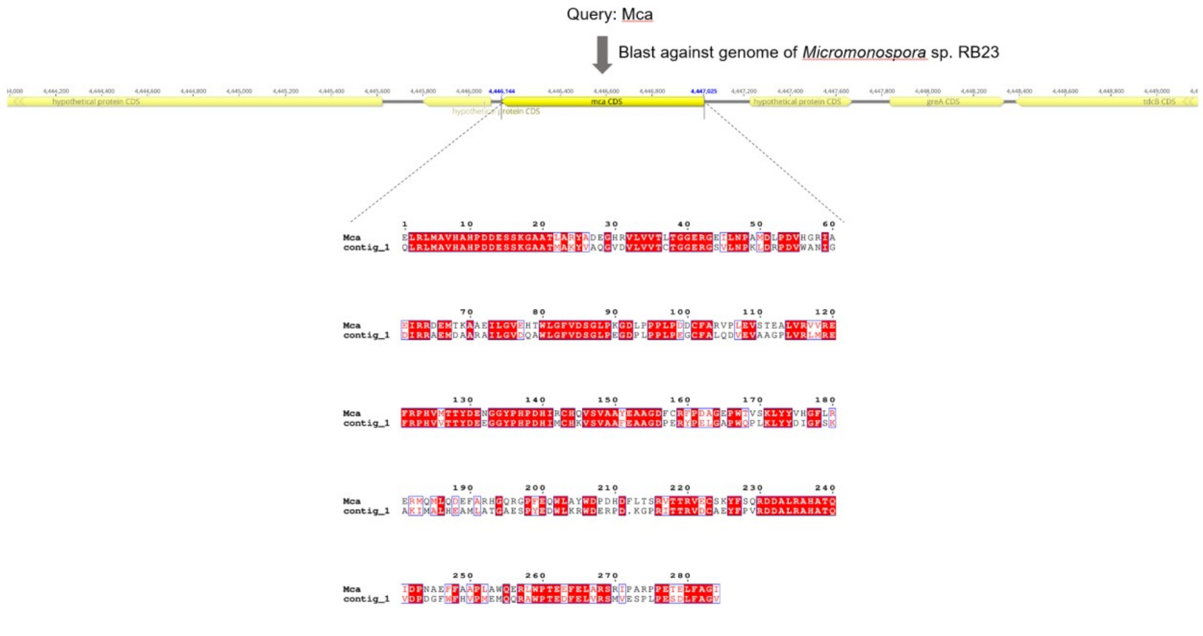

Figure S9. Blast result of putative *mca*-like gene in the genome of *Micromonospora* sp. RB23 using mycothiol S-conjugated amidase (Mca) from *Mycobacterium tuberculosis H37Rv* as query.[20] Sequence alignment of Mca and candidate contig_1 from RB23 using Clustal Omega showed overall identity of 59.5% and the figure was generated by ESPript 3.0.[21]

Table S3. BGCs annotation using antiSMASH 8.0 with relaxed setting

Region	Type	Confidence	Most similar known cluster	Comment
Region 1	terpene-precursor	Low	CDA1b/CDA2a/CDA2b/CDA3a/CDA3b/CDA4a/CDA4b	NRP:Lipopeptide:Ca+-dependent lipopeptide
Region 2	NRPS	Low	vazabitide A	NRP
Region 3	terpene-precursor			
Region 4	terpene			
Region 5	RiPP-like,terpene	Low	lymphostin/neolymphostinol B/lymphostinol/neolymphostin b	Polyketide+NRP
Region 6	NRPS-like,T1PKS,NRP-metallophore,NRPS	Low	kedarcidin	NRP+Polyketide:Iterative type I polyketide+Polyketide:Enediyne type I polyketide
Region 7	T1PKS	Low	pyrrolomycin A/pyrrolomycin B/pyrrolomycin C/pyrrolomycin D	Polyketide
Region 8	lanthipeptide-class-iii	High	SapB	RiPP:Lanthipeptide
Region 9	NRPS,T1PKS,PKS-like			
Region 10	NI-siderophore	High	legonoxamine A/desferrioxamine B/legonoxamine B	Other
Region 11	T2PKS	Low	pradimicin-A	Polyketide
Region 12	terpene			
Region 13	terpene-precursor			
Region 14	terpene			
Region 15	terpene	Low	isorenieratene	Terpene
Region 16	T3PKS	High	loseolamycin A1/loseolamycin A2	Polyketide

Table S4. Proteins encoded in *mcs* BGC and blast result against NCBI Refseq database

**Name**	**Size (aa)**	**Identity**	**Covery**	**Positives**	**Sequence similarity in NCBI (Protein, Origin)**	**Acc. No.**
McsA	265	81	100	85	Thioesterase II family protein [*Micromonospora ureilytica*]	WP_196925832.1
McsB	2654	78	99	82	Type I polyketide synthase [*Micromonospora ureilytica*]	WP_196925833.1
McsC	2226	86	100	90	Type I polyketide synthase [*Micromonospora ureilytica*]	WP_196925834.1
McsD	579	96	100	98	NAD(P)/FAD-dependent oxidoreductase [*Micromonospora ureilytica*]	WP_196925835.1
McsE	586	95	100	97	NAD(P)/FAD-dependent oxidoreductase [*Micromonospora ureilytica*]	WP_196925836.1
McsF	555	97	100	98	NAD(P)/FAD-dependent oxidoreductase [*Micromonospora ureilytica*]	WP_196925837.1
McsG	590	86	100	89	NAD(P)/FAD-dependent oxidoreductase [*Micromonospora ureilytica*]	WP_196925838.1
McsH	196	95	100	97	TetR family transcriptional regulator [*Micromonospora ureilytica*]	WP_124822745.1
McsI	378	93	100	95	Acyl-CoA dehydrogenase family protein [*Micromonospora ureilytica*]	WP_196925839.1
McsJ	526	89	99	91	AMP-binding protein [*Micromonospora ureilytica*]	WP_196925840.1
McsK	181	92	95	94	Flavin reductase family protein [*Micromonospora ureilytica*]	WP_124822747.1
McsL	477	91	100	95	DHA2 family efflux MFS transporter permease subunit [*Micromonospora ureilytica*]	WP_196925841.1
McsM	110	94	61	97	MULTISPECIES: hypothetical protein [*Micromonospora*]	WP_196925842.1
McsN	153	91	99	94	Methyltransferase family protein [*Micromonospora ureilytica*]	WP_196925843.1
McsO	184	96	100	97	TetR family transcriptional regulator [*Micromonospora ureilytica*]	WP_196925844.1
McsP	92	96	100	98	Acyl carrier protein [*Micromonospora ureilytica*]	WP_196925845.1
McsQ	451	95	100	97	MULTISPECIES: NAD(P)/FAD-dependent oxidoreductase [*Micromonospora*]	WP_124822751.1

Table S5. NMR spectroscopic data (500 MHz) of pyrrolomycin K (**3**) and pyrrolomycin L (**5**)

**3**			**5**	
Acetone-d_6			DMSO-d_6	
position	δ_C, type	δ_H, *(J in Hz)*	δ_C, type	δ_H *(J in Hz)*
1				
2	128.6, C		135.4, C	
3	120.1, CH	6.96(s)	117.2, C	
4	110.1, C		114.4, C	
5	125.1, C		122.7, C	
6	186.1, C		180.5, C	
7	122.7, C		121.1, C	
8	159.7, C		156.9, C	
9	119.7, CH	7.05 (d, 8.7)	121.3, CH	6.82 (1, d, 8.8)
10	134.9, CH	7.52 (dd, 2.8, 8.8)	132.1, CH	7.29 (1, dd, 8.8, 3.0)
11	123.5, C		127.7, C	
12	131.1, CH	7.73 (d, 2.8)	131.3, CH	8.04 (1, d, 2.8)
13	34.1, CH_3	3.96 (s)	36.4, CH_2	3.31 (1, dd, 4.7, 13.3); 2.99 (1, dd, 8.8, 13.0)
14			52.2, CH	4.08 (1, m)
15			172.7, C	
16				8.12 (1, d, 7.5)
17			169.0, C	
18			22.4, CH_3	1.8 (3, s)

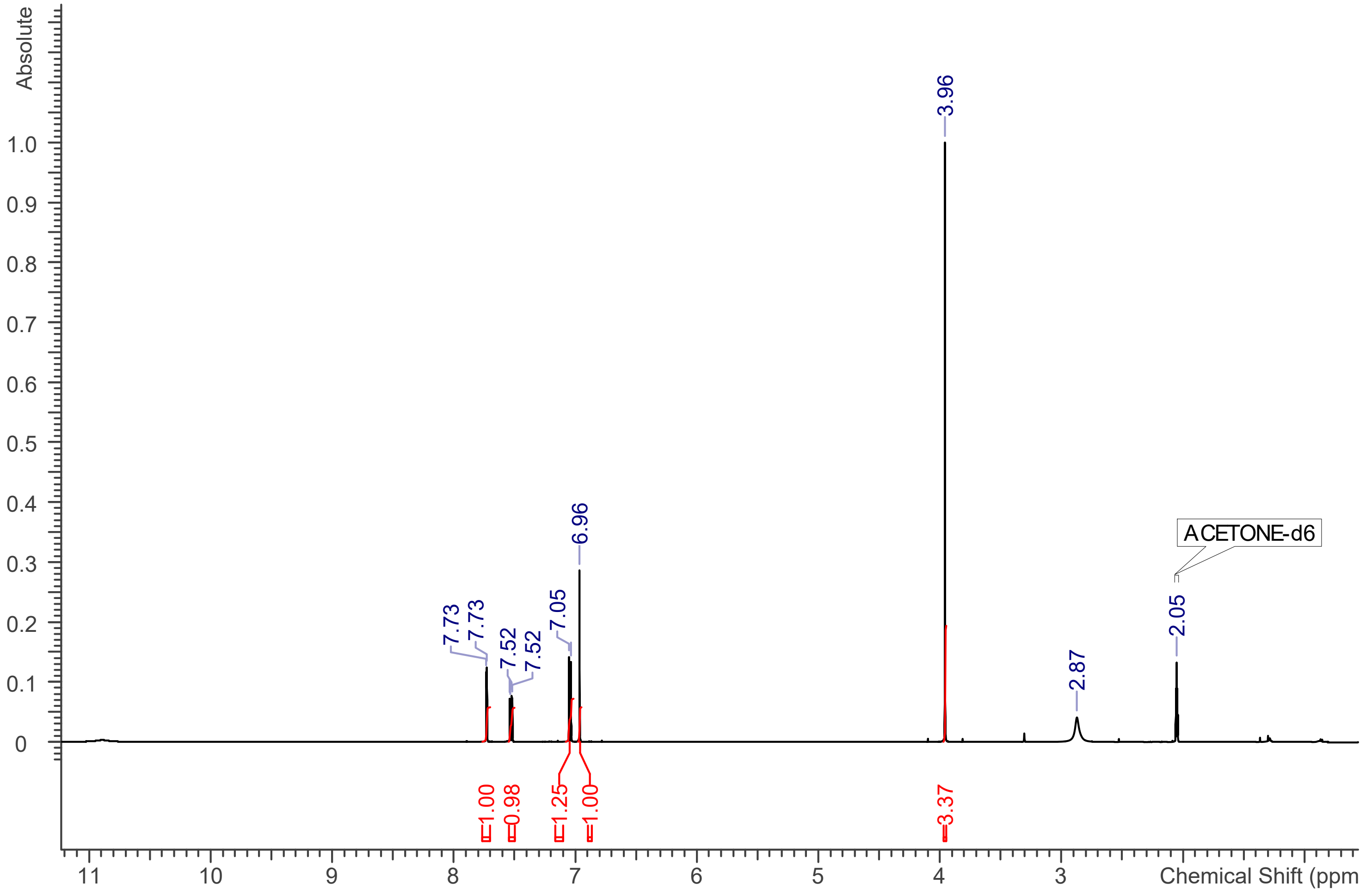

Figure S10. ^{1}H NMR spectrum of pyrrolomycin K (**3**) in acetone-*d*$_6$

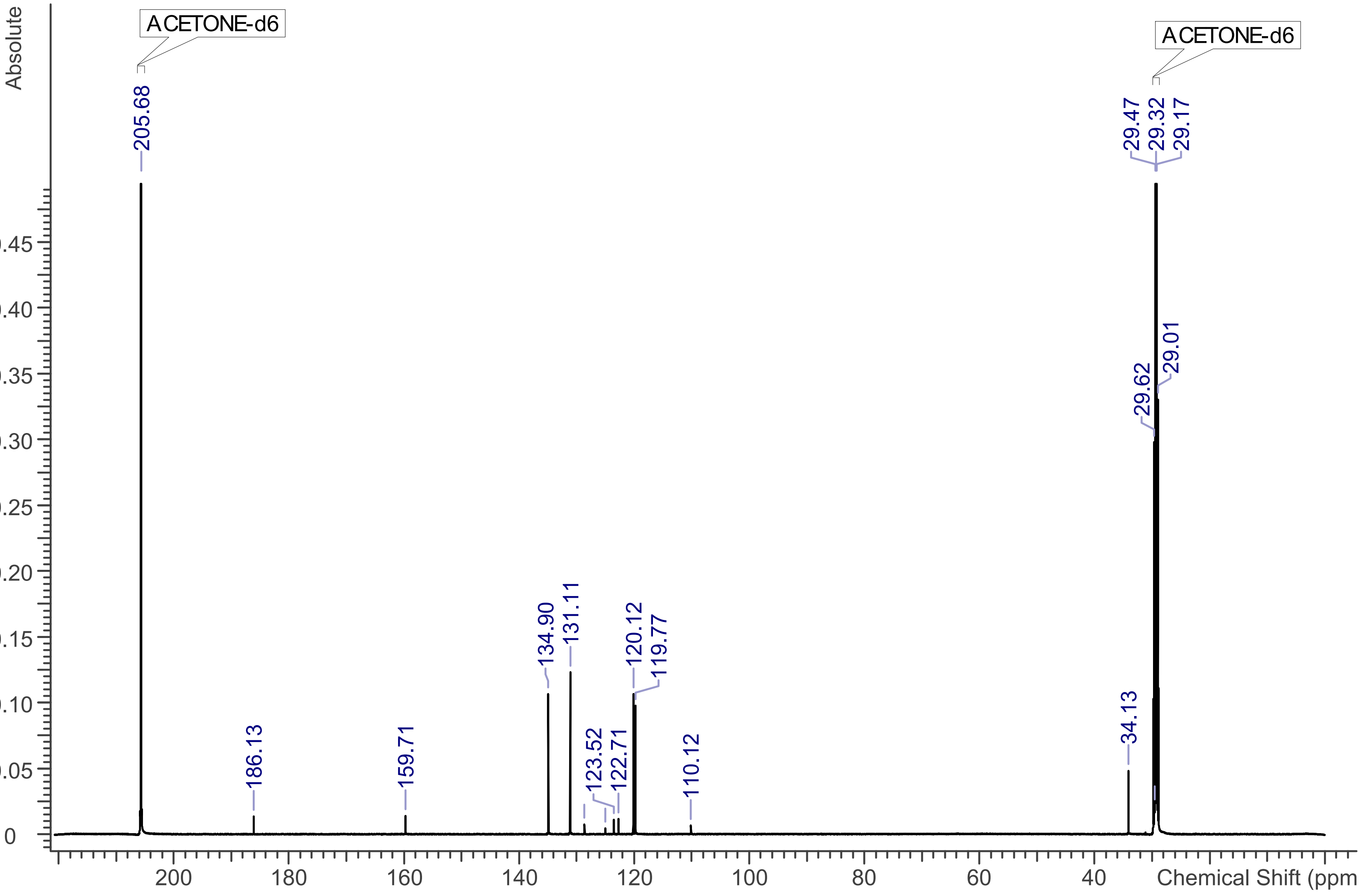

Figure S11. ^{13}C NMR spectrum of pyrrolomycin K (**3**) in acetone-d_6

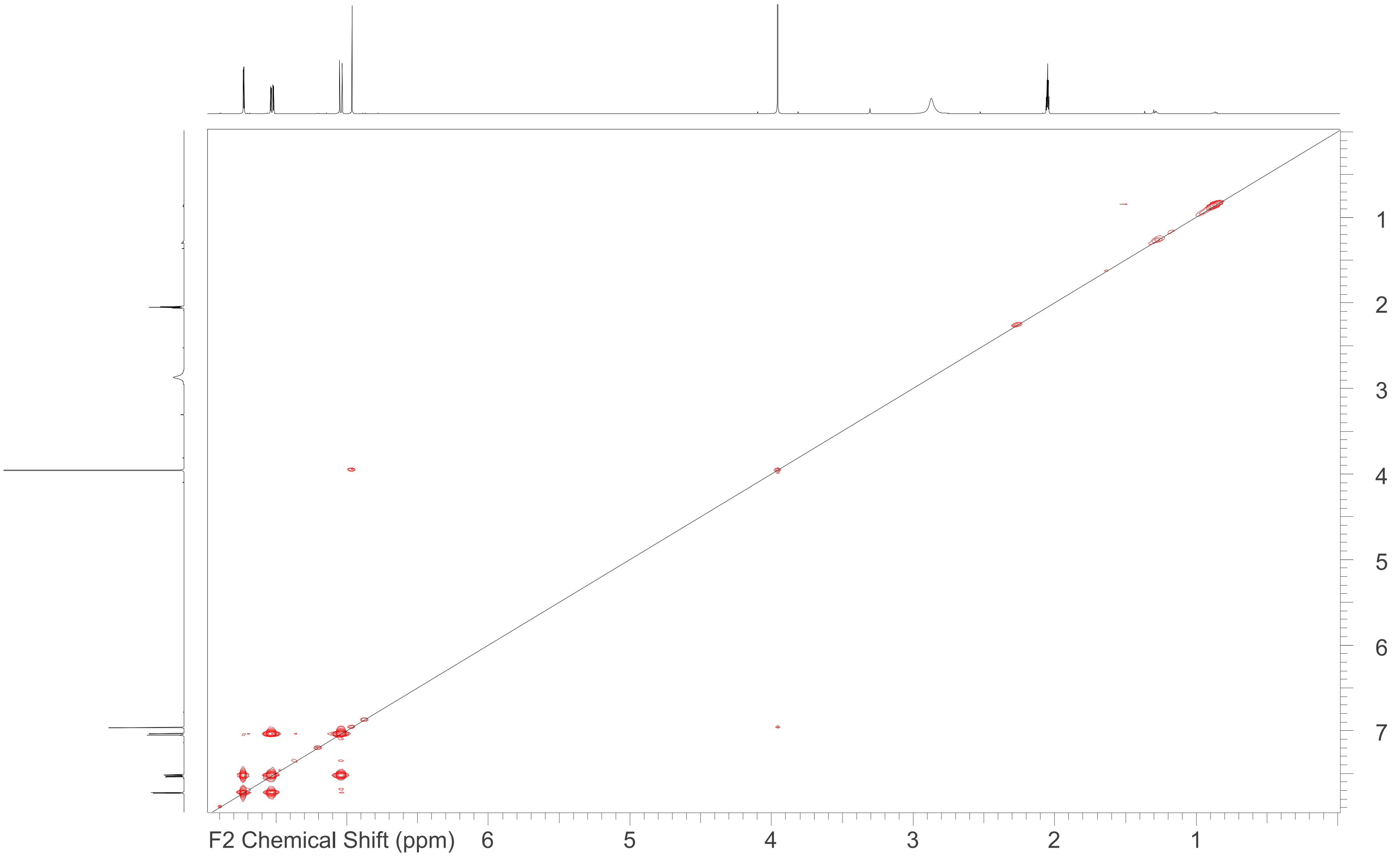

Figure S12. COSY spectrum of pyrrolomycin K (**3**) in acetone-d_6

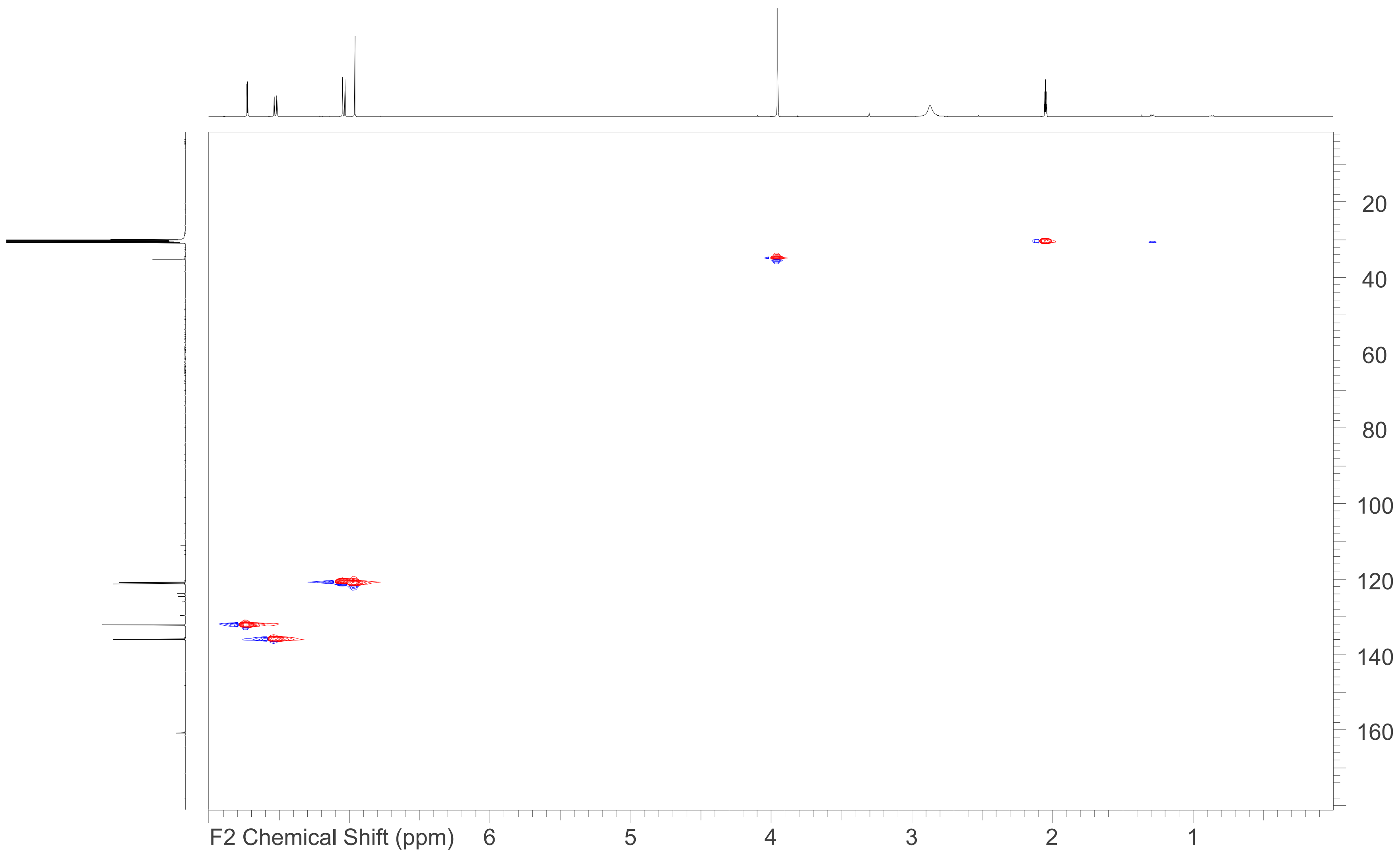

Figure S13. HSQC spectrum of pyrrolomycin K (**3**) in acetone-d_6

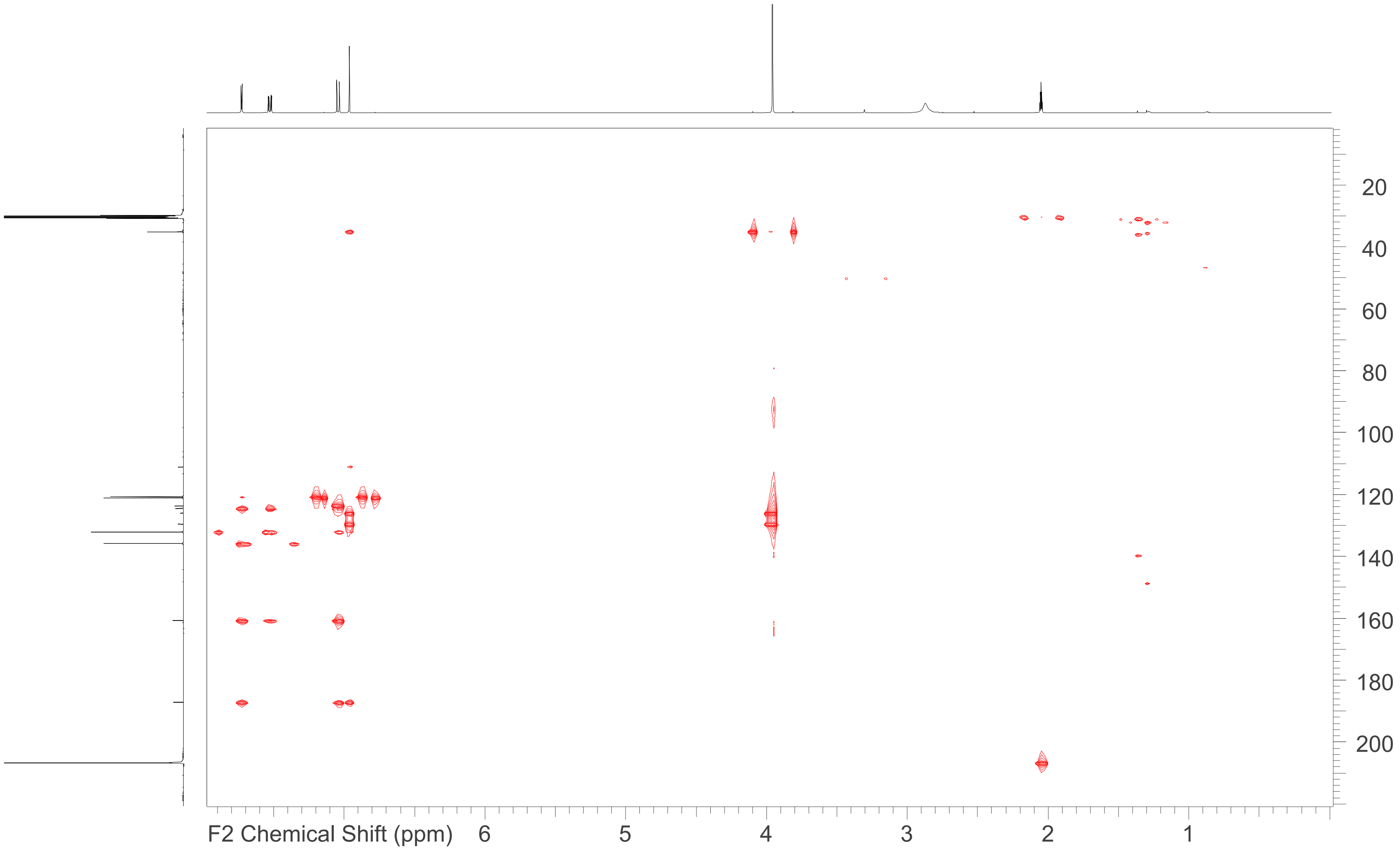

Figure S14. HMBC spectrum of pyrrolomycin K (**3**) in acetone-d_6

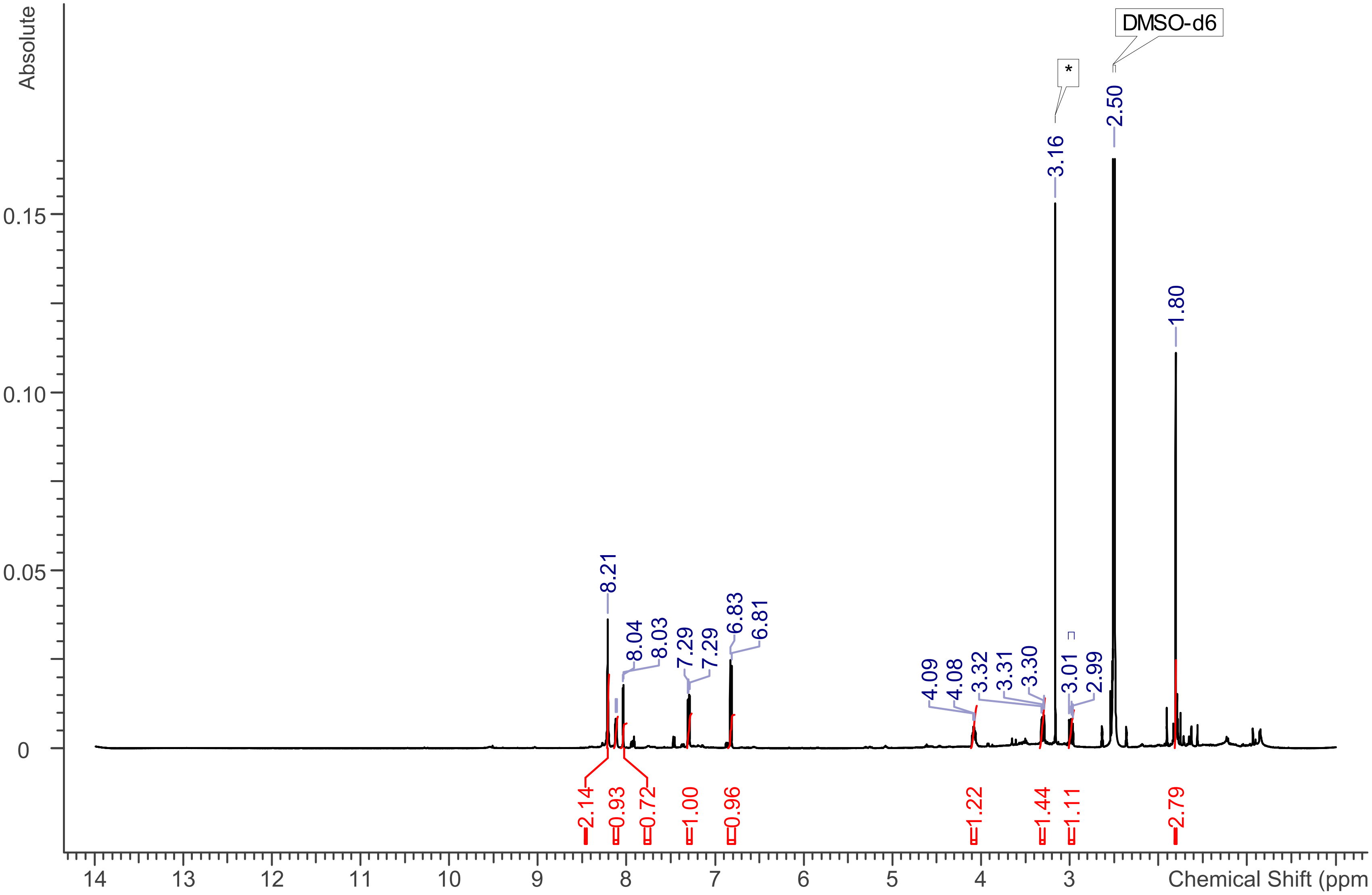

Figure S15. ^{1}H NMR spectrum of pyrrolomycin L (**5**) in DMSO-d_6 (* impurity signal from MeOD)

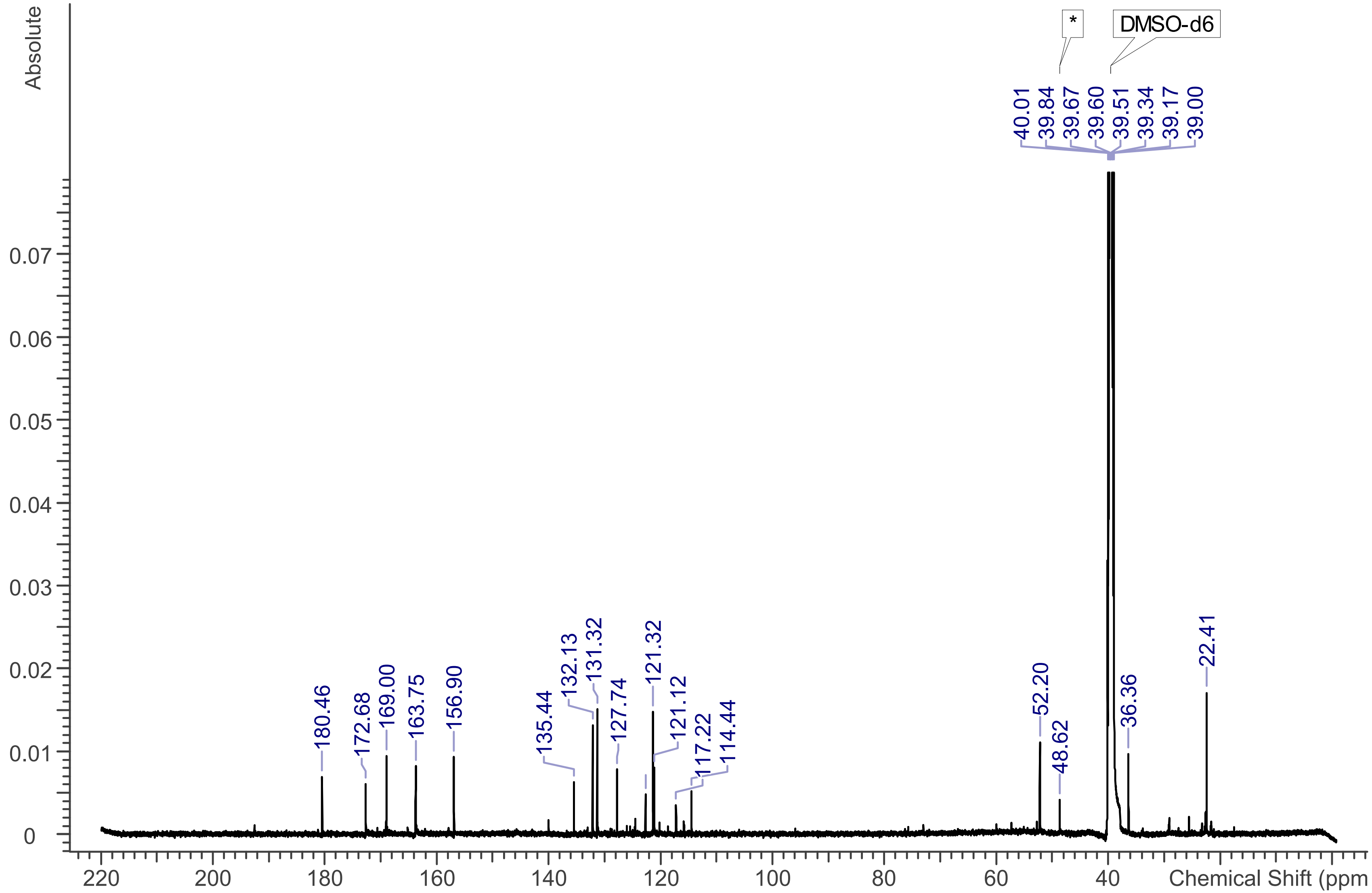

Figure S16. ^{13}C NMR spectrum of pyrrolomycin L (**5**) in DMSO-d_6 (* impurity from MeOD)

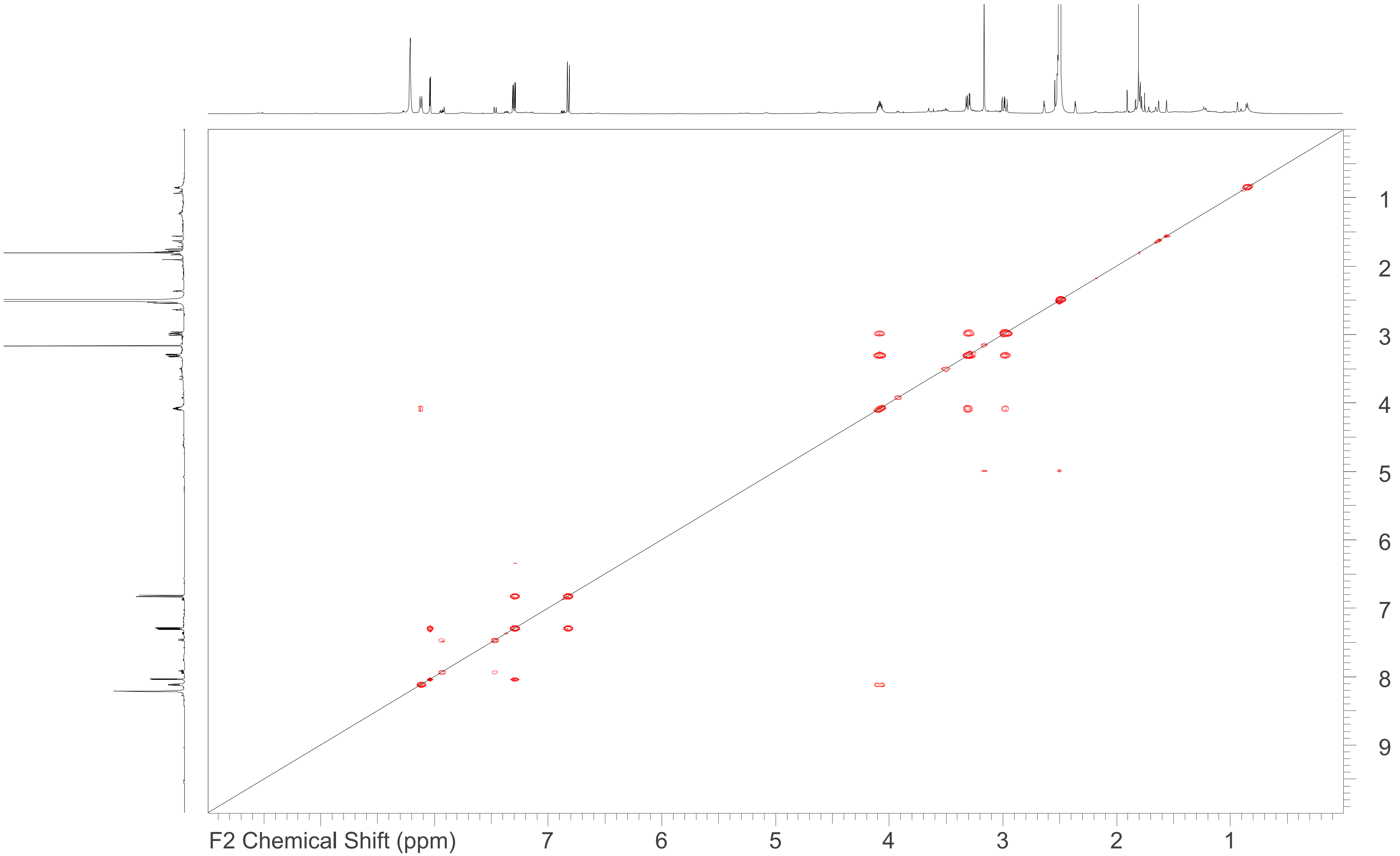

Figure S17. COSY spectrum of pyrrolomycin L (**5**) in DMSO-d_6

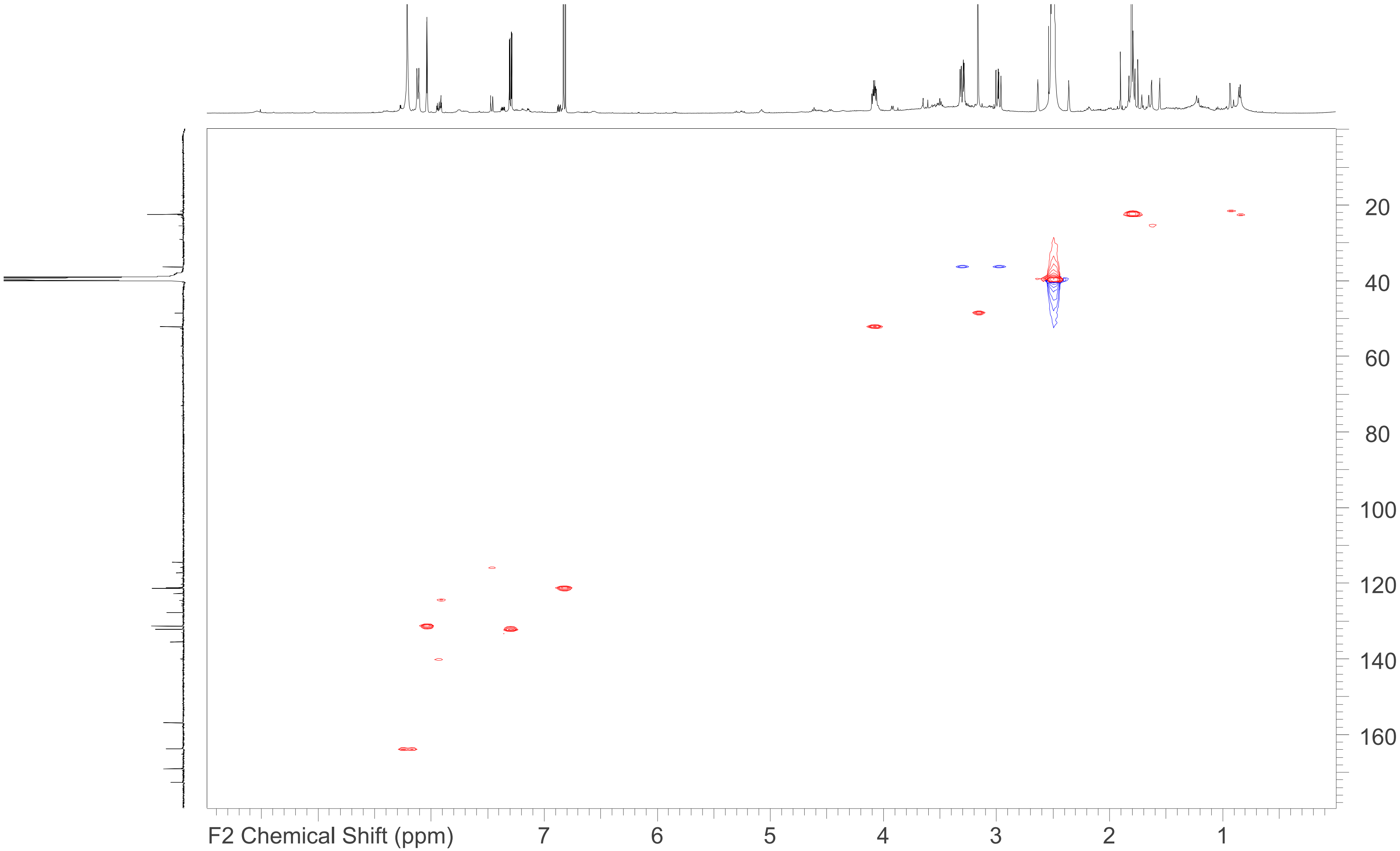

Figure S18. HSQC spectrum of pyrrolomycin L (**5**) in DMSO-d_6

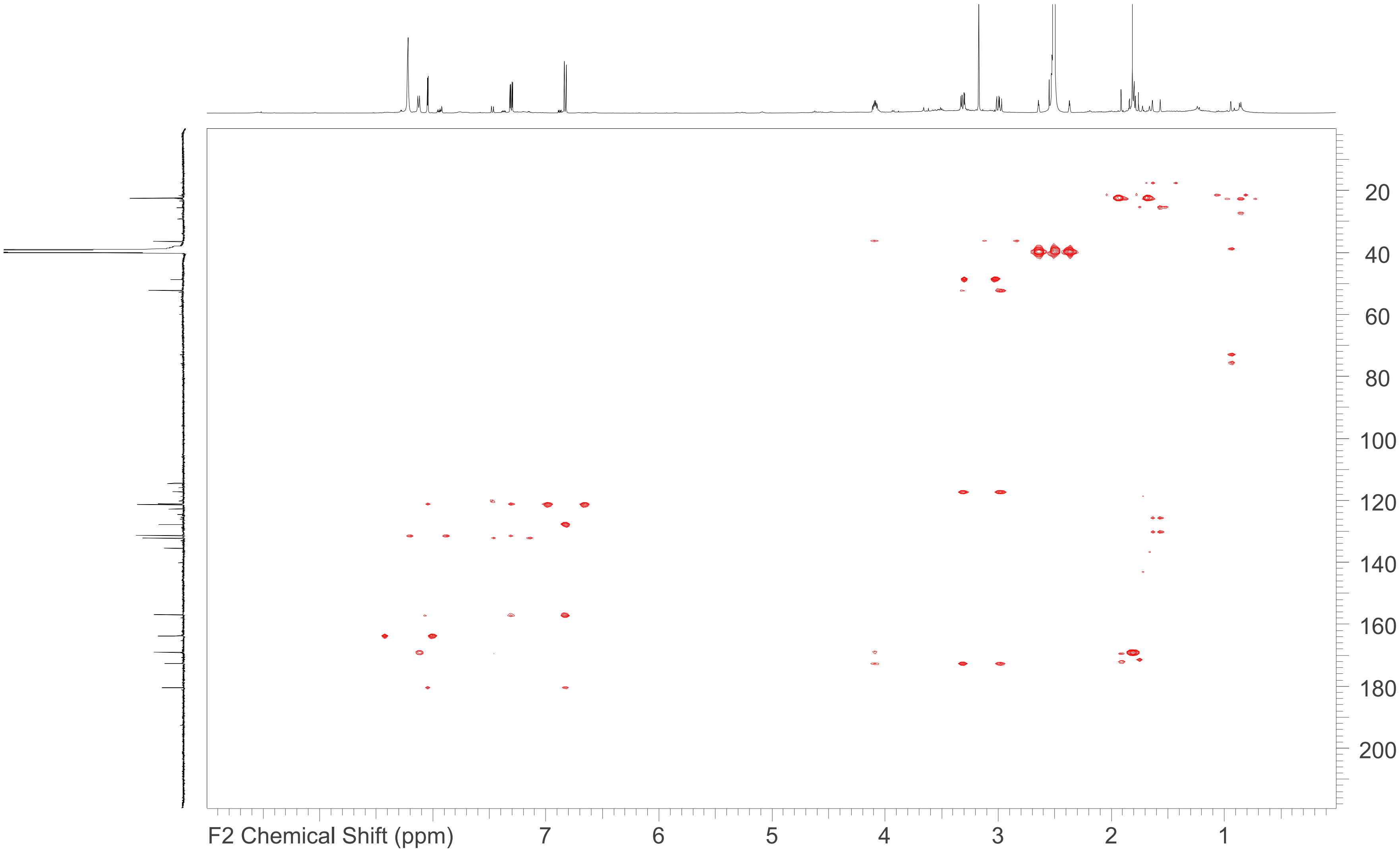

Figure S19. HMBC spectrum of pyrrolomycin L (**5**) in DMSO-*d*$_6$

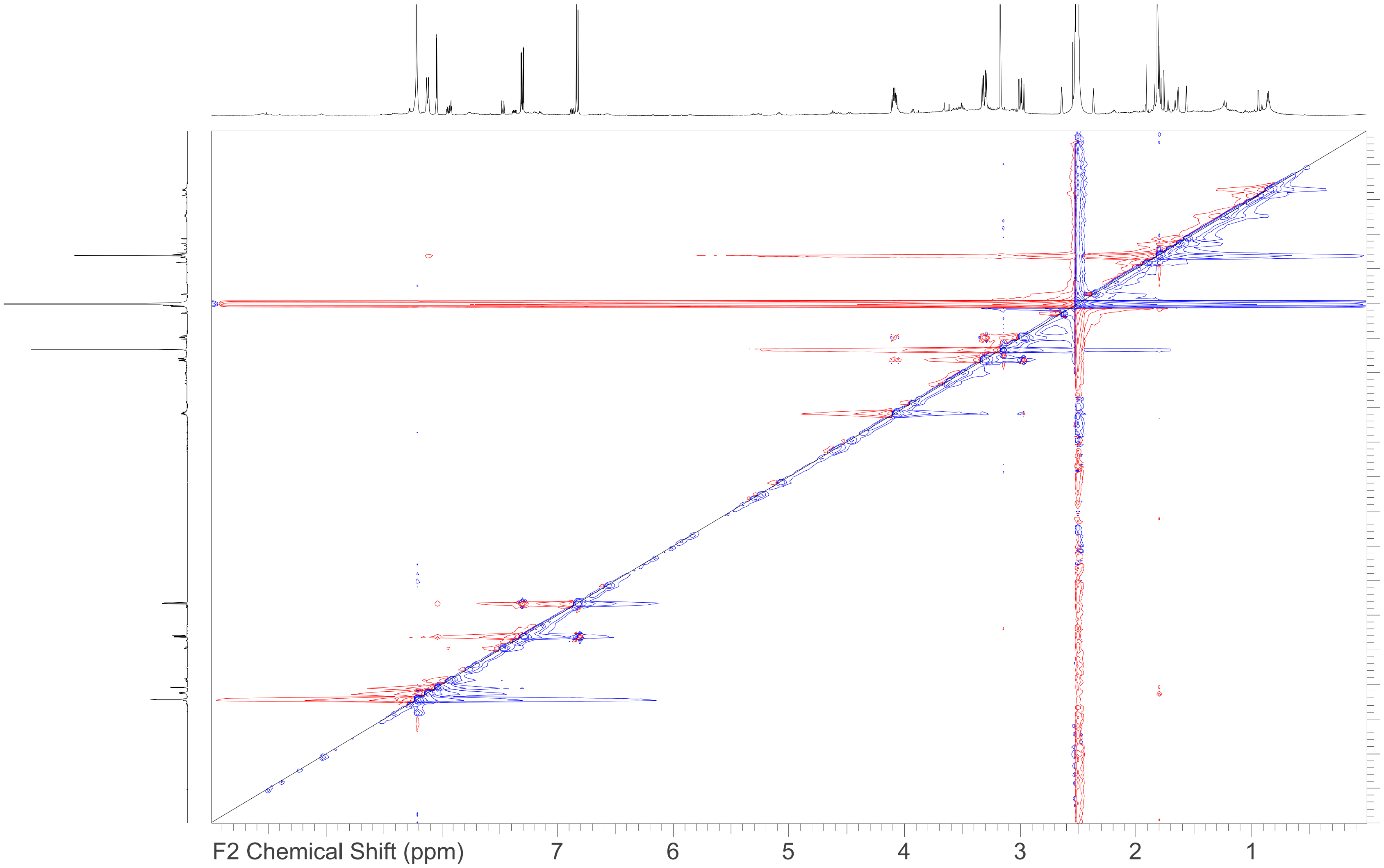

Figure S20. NOESY spectrum of pyrrolomycin L (**5**) in DMSO-d_6

Table S6. Antimicrobial activity results (MIC, µg/mL)

	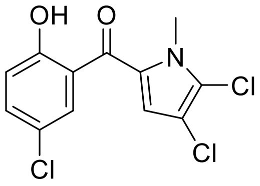	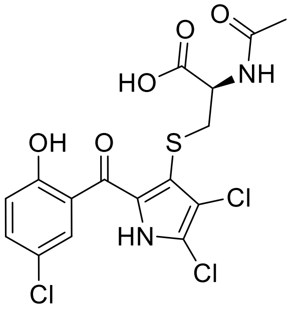	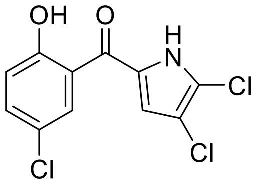		
Compound	**3**	**5**	**2**[22]	Vancomycin	Amphotericin B
C. albicans DSM-1665	> 64	> 64			0.125
E. coli ΔtolC	> 64	64		8	
S. aureus ATCC 29213	> 64	64		1	
S. aureus 209-P			0.4		
S. aureus (resistant)			0.8		

Reference

(1) Lefort, V.; Desper, R.; Gascuel, O. FastME 2.0: A Comprehensive, Accurate, and Fast Distance-Based Phylogeny Inference Program. *Mol Biol Evol.* **2015**, *32* (10), 2798-2800.

(2) Farris, J. S. Estimating Phylogenetic Trees from Distance Matrices. *Am. Nat.* **1972**, *106* (951), 645-668.

(3) Meier-Kolthoff, J. P.; Goker, M. TYGS is an automated high-throughput platform for state-of-the-art genome-based taxonomy. *Nat Commun.* **2019**, *10* (1), 2182.

(4) Vind, K.; Maffioli, S.; Fernandez Ciruelos, B.; Waschulin, V.; Brunati, C.; Simone, M.; Sosio, M.; Donadio, S. N-Acetyl-Cysteinylated Streptophenazines from *Streptomyces*. *J. Nat. Prod.* **2022**, *85* (5), 1239-1247.

(5) Nakashima, T.; Kimura, T.; Miyano, R.; Matsuo, H.; Hirose, T.; Kimishima, A.; Nonaka, K.; Iwatsuki, M.; Nakanishi, J.; Takahashi, Y.; et al. Nanaomycin H: A new nanaomycin analog. *J Biosci Bioeng.* **2017**, *123* (6), 765-770.

(6) NORIO EZAKI, T. S., MASAO KOYAMA, TOMIZO NIWA, MICTUO KOJIMA, SHIGEHARU INOUYE, TATSUO ITO, TARO NIIDA. NEW CHLORINATED NITRO-PYRROLE ANTIBIOTICS, PYRROLOMYCIN A AND B (SF-2080 A AND B). *J. Antibiot.* **1981**, *34* (10), 1363-1365.

(7) Ezaki, N.; Koyama, M.; Shomura, T.; Tsuruoka, T.; Inouye, S. Pyrrolomycins C, D and E, new members of pyrrolomycins. *J. Antibiot. (Tokyo)* **1983**, *36* (10), 1263-1267.

(8) Charan, R. D.; Schlingmann, G.; Bernan, V. S.; Feng, X.; Carter, G. T. Additional pyrrolomycins from cultures of *Streptomyces fumanus*. *J. Nat. Prod.* **2005**, *68* (2), 277-279.

(9) Charan, R. D.; Schlingmann, G.; Bernan, V. S.; Feng, X.; Carter, G. T. Dioxapyrrolomycin Biosynthesis in *Streptomyces fumanus*. *J. Nat. Prod.* **2006**, *69* (1), 29-33.

(10) NORIO EZAKI, M. K., YOSHIO KODAMA, TAKASHI SHOMURA, KUMIKO TASHIRO, TAKASHI TSURUOKA, SHIGEHARU INOUYE, SHIN-ICHIRO SAKAI. PYRROLOMYCINS F1 F2a, F2b AND F3, NEW METABOLITES PRODUCED BY THE

ADDITION OF BROMIDE TO THE FERMENTATION. *J. Antibiot.* **1983**, *36* (11), 1431-1438.

(11) Nowak-Thompson, B.; Gould, S. J.; Loper, J. E. Identification and sequence analysis of the genes encoding a polyketide synthase required for pyoluteorin biosynthesis in *Pseudomonas fluorescens* Pf-5. *Gene.* **1997**, *204* (1-2), 17-24.

(12) Dufour, C.; Wink, J.; Kurz, M.; Kogler, H.; Olivan, H.; Sable, S.; Heyse, W.; Gerlitz, M.; Toti, L.; Nusser, A.; et al. Isolation and structural elucidation of armeniaspirols A-C: potent antibiotics against gram-positive pathogens. *Chemistry (Easton).* **2012**, *18* (50), 16123-16128.

(13) Hughes, C. C.; Prieto-Davo, A.; Jensen, P. R.; Fenical, W. The marinopyrroles, antibiotics of an unprecedented structure class from a marine *Streptomyces* sp. *Org. Lett.* **2008**, *10* (4), 629-631.

(14) Flatt, P. M.; Wu, X.; Perry, S.; Mahmud, T. Genetic insights into pyralomicin biosynthesis in *Nonomuraea spiralis* IMC A-0156. *J. Nat. Prod.* **2013**, *76* (5), 939-946.

(15) Wu, Q.; Liang, J.; Lin, S.; Zhou, X.; Bai, L.; Deng, Z.; Wang, Z. Characterization of the biosynthesis gene cluster for the pyrrole polyether antibiotic calcimycin (A23187) in *Streptomyces chartreusis* NRRL 3882. *Antimicrob Agents Chemother.* **2011**, *55* (3), 974-982.

(16) Yi, D.; Acharya, A.; Gumbart, J. C.; Gutekunst, W. R.; Agarwal, V. Gatekeeping Ketosynthases Dictate Initiation of Assembly Line Biosynthesis of Pyrrolic Polyketides. *J. Am. Chem. Soc.* **2021**, *143* (20), 7617-7622.

(17) Mantovani, S. M.; Moore, B. S. Flavin-linked oxidase catalyzes pyrrolizine formation of dichloropyrrole-containing polyketide extender unit in chlorizidine A. *J. Am. Chem. Soc.* **2013**, *135* (48), 18032-18035.

(18) Witte, S. N. R.; Hug, J. J.; Geraldy, M. N. E.; Muller, R.; Kalesse, M. Biosynthesis and Total Synthesis of Pyrronazol B: a Secondary Metabolite from *Nannocystis pusilla*. *Chemistry (Easton)* **2017**, *23* (63), 15917-15921.

(19) Meiser, P.; Weissman, K. J.; Bode, H. B.; Krug, D.; Dickschat, J. S.; Sandmann, A.; Muller, R. DKxanthene biosynthesis--understanding the basis for diversity-oriented synthesis in myxobacterial secondary metabolism. *Chem Biol.* **2008**, *15* (8), 771-781.

(20) Rawat, M.; Uppal, M.; Newton, G.; Steffek, M.; Fahey, R. C.; Av-Gay, Y. Targeted mutagenesis of the *Mycobacterium smegmatis* mca gene, encoding a mycothiol-dependent detoxification protein. *J. Bacteriol.* **2004**, *186* (18), 6050-6058.

(21) Robert, X.; Gouet, P. Deciphering key features in protein structures with the new ENDscript server. *Nucleic Acids Res.* **2014**, *42* (Web Server issue), W320-324.

(22) Durham, D. G.; Hughes, C. G.; Rees, A. H. The Chlorination of Pyrroles. Part III. *Can. J. Chem.* **1972**, *50* (19), 3223-3228.
